# Bi-Functional Extension on Heterogeneous ORR/OER Catalysis with 2D Materials for Li-O_2_ Batteries

**DOI:** 10.1007/s40820-026-02235-3

**Published:** 2026-05-28

**Authors:** Guoliang Zhang, Han Yu, Ruonan Yang, Yuqi Fan, Ning Wang, Zhanhu Guo, Feng Dang

**Affiliations:** 1 Key Laboratory for Liquid-Solid Structure Evolution and Processing of Materials (Ministry of Education), Shandong University, Jinan, 250061 People’s Republic of China; 2 College of Geography and Environment, Shandong Normal University, Jinan, 250358 People’s Republic of China; 3 State Key Laboratory of Marine Resource Utilization in South China Sea, Hainan University, Haikou, 570228 People’s Republic of China; 4 Department of Mechanical and Construction Engineering, Northumbria University, Newcastle Upon Tyne, NE1 8ST UK

**Keywords:** Li–O_2_ batteries, Two-dimensional materials, Cathode catalysts, Activation strategies, Anisotropic catalytic surface

## Abstract

In the integration of heterogeneous catalysis with two-dimensional (2D) materials, Li–O_2_ batteries serve as valuable research prototypes, particularly for investigating the efficient oxygen reduction/evolution reaction in multiphase environments. However, the advantages and limitations associated with the unique anisotropy and electronic properties of 2D materials in Li–O_2_ batteries (LOBs) remain unclear. This Review provides a comprehensive overview of 2D cathode catalysts for LOBs, including graphene, transition metal oxides/hydroxides, dichalcogenides, and metal carbides, together with their corresponding activation engineering strategies. More specifically, we thoroughly examine the pivotal role of anisotropic catalytic properties on the large surface areas and terminal edge active sites of 2D cathode catalysts. The correlations are analyzed between 2D material design and catalytic mechanisms in LOBs, particularly in adsorption strength of intermediates, electronic structure and discharge product. Additionally, the expanded applications of 2D materials in components such as lithium anode protection and rapid lithium-ion transport are also briefly discussed by leveraging their favorable physicochemical properties. Finally, the challenges and future directions for the development and application of 2D materials are summarized and discussed, which is expected to unlock the full potential of LOBs batteries as next-generation energy storage technologies.

## Highlights

- This review systematically summarizes the catalytic performance of two-dimensional (2D) materials in Li–O_2_ batteries, together with activation strategies spanning point, line, plane, and bulk dimensions.
- The catalytic mechanisms and key descriptors of 2D materials are analyzed, including the adsorption strength of intermediates, electronic structure, and product evolution.
- The multifunctional roles of 2D materials in Li–O_2_ batteries are discussed, including separators, electrolyte additives, and lithium anode protection.

## Graphical abstract

**Figure:**
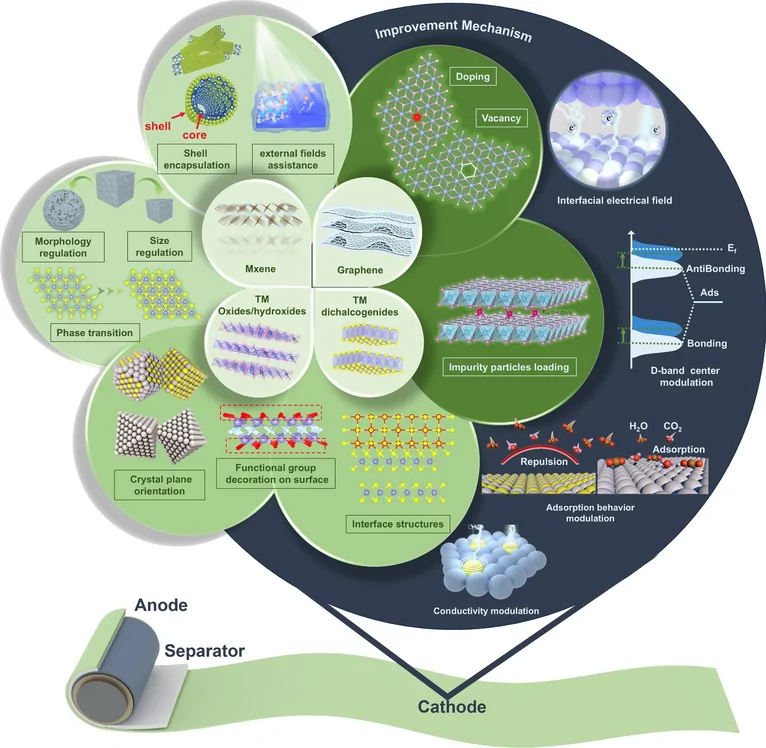

## Introduction

Two-dimensional (2D) nanomaterials flourished with the successful exfoliation of graphene, an atomically thin carbon material [1–3]. Subsequently, other classes of ultrathin 2D nanomaterials have been extensively studied, including, but not limited to, phosphorene [4], graphitic carbon nitride (g-C_3_N_4_) [5, 6], layered oxides/hydroxides [7, 8], MXenes [9, 10], and transition metal dichalcogenides (TMDs) [11, 12]. 2D materials feature an ultrathin thickness of monolayer or several atomic layers connecting with unique bonding interactions. There are strong covalent bonds within the atomic planes of 2D nanomaterials, whereas weak van der Waals force interactions exist between the stacked layers. This structural anisotropy endows 2D materials with physicochemical properties distinct from their bulk counterparts, including a large surface area, abundant exposed active sites, uniform surface chemistry, and tunable electronic structure and catalytic activity [13]. Importantly, understanding such anisotropy is crucial for elucidating and optimizing reversible catalytic reactions.

Li–O_2_ batteries (LOBs) are one of valuable research prototypes integration of reversible catalytic processes with 2D catalysts [12, 14]. In LOBs, it experiences two main processes: oxygen reduction reaction (ORR) in discharging process and oxygen evolution reaction (OER) in charging process. During discharge, Li ions stripping from anode move to cathode combined with reduced oxygen molecules forming solid products Li_2_O_2_ and depositing on the cathode. The charging process proceeds in reverse with Li ions diffusing back and O_2_ released, which was proved by Bruce et al. using mass spectrometry technology in 2006 [15, 16]. After that, primary researches concentrate on revealing the detailed cathode reaction mechanism during Li_2_O_2_ formation and decomposition to promote the reversibility of LOBs [17]. It is deeply recognized that the catalysts highly dominate the electrochemical performance for LOBs, in reaction mechanism difference, rate performance, discharge capacity, and cycling stability [14, 15].

2D materials have attracted the sights as electrocatalysts, which greatly rely on the electronic and structural properties [13, 18]. However, it has to be said that most pristine 2D catalysts are inferior in catalytic performance resulting from low density of electronic states [1, 19, 20]. Fortunately, the electronic properties of 2D materials can be easily activated by activation engineering, becoming the research hot spots to design highly active 2D materials in improving the electrochemical performance [21, 22]. Based on the dimension of the modulation area, the activation engineering can be categorized as follows: (1) *Point activation engineering*, also known as small particle or even single atomic level modulation, includes vacancies [23, 24], heteroatomic doping [3, 20, 25], and impurity particles loading [26, 27]. The “point” usually serves as the catalytic centers, which can modulate the local electronic states, reactant adsorption/desorption properties, and selectivity. (2) *Line and plane activation engineering* refer to the one- or two-dimensional scale designs, including but not limited to the line defects [12], interface structures [28, 29], functional group decoration on surface [10, 30], and crystal plane orientation [31, 32]. These methods can efficiently tune the electronic states of 2D materials and ultimately affect the electrochemical performance in the catalytic reactions [33]. (3) *Bulk activation engineering* focuses on the three-dimensional directions, such as morphology and size regulation [34, 35], shell encapsulation [5, 36], phase transition [37], or external fields assistance [38, 39]. Compared with the above two engineering, bulk activation will induce more pronounced changes in intrinsic properties or catalytic reactions.

**Fig. 1:**
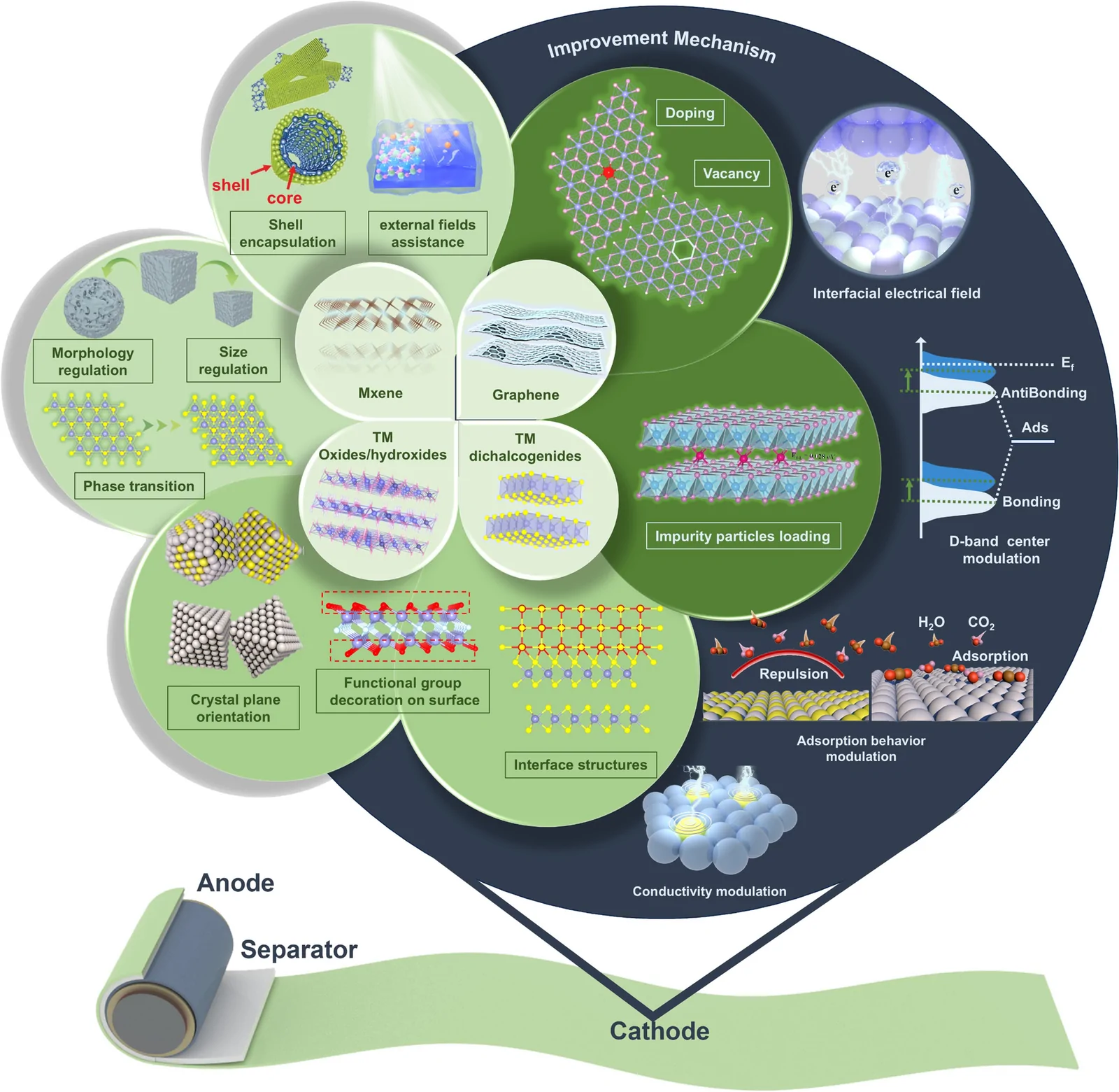
Schematic illustration of typical 2D materials, modification methods, and mechanisms in LOBs [185, 186, 247]. Copyright 2024, Elsevier. Copyright 2022, John Wiley and Sons. Copyright 2021, John Wiley and Sons

With the development of highly efficient cathode catalysts, it is gradually acknowledged that the modification and cooperation of anode, electrolyte and separator are of greater importance for further improving the overall performance, including cycle stability, safety, and resistance to complex environments [40, 41]. Talking about lithium metal, the uncontrollable morphological changes of Li anode during repeated discharge/charge processes have long jeopardized the battery performance [15, 42]. The generated Li dendrites can easily penetrate the separator, leading to the internal short circuit. Meanwhile, the morphology changes of Li metal constantly destroy the solid–electrolyte interface (SEI) films and consume fresh Li and electrolyte. Recently, 2D materials have exerted their functions to support the battery system due to their highly valuable physicochemical properties [43–45]. The Li dendrite can be suppressed by 2D materials with high mechanical strength [46]. Furthermore, 2D materials would modulate the lithium-ion flux and uniformize the deposition of lithium, stabilizing the environment in the battery system [47–49]. Given the great potential of LOBs and multiple functions of 2D materials, a timely review about combining these two aspects is essential. This review highlights the irreplaceable role of 2D materials in LOBs and summarizes recently reported progress (Fig. 1). We firstly introduce the working mechanism and main challenges in the LOBs, especially for seeking highly efficient catalysts. Then, according to the different activation engineering dimensions, we classified the different types of 2D materials as cathode catalysts and discussed the improved electrocatalytic performance, aiming to clarify the catalytic mechanisms of activated 2D materials in LOBs. The mechanistic perspective across 2D materials was discussed. Subsequently, in view of the issues in the anode, separator, and electrolyte, a concise analysis was made for the extended applicability of 2D materials from fundamental physicochemical perspective. Finally, we address the prevailing challenges and prospects with 2D material for the future development of the LOBs.

## Working Principle and Challenge in Li–O_2_ Batteries

As a promising energy storage system with an exceptionally high theoretical energy density of 3500 Wh kg^−1^, the practical application of LOBs has been restricted by many technical challenges, including low round-trip efficiency, degraded capacity and cyclability. These major challenges are closely related to the function of each component of LOBs. Until now, considerable research efforts have been devoted into revealing the working principle, thus enhancing the corresponded performance. Basically, this section reviews the cathode reaction mechanisms and challenges in LOBs, and the role of other components.

### Fundamental Understanding in Cathode Reaction

Understanding the reaction mechanisms of LOBs bears profound guiding implications for the design of the cathode. Considerable research efforts have been devoted to ORR and OER processes in LOBs, demonstrating the circumstance of single-electron [50], two-electron [51], and four-electron [52, 53] reduction of O_2_ to LiO_2_, Li_2_O_2_, and Li_2_O products, respectively. Actually, the reaction path greatly depends on the competition effects of various factors, e.g., electrolyte properties [54, 55], electrolyte additives [56], and electrocatalysts [57, 58]. General speaking, it is currently hard to set a consensual product target considering the complex reactivity in LOBs. Take the widely studied Li_2_O_2_ as example, the formation process can be described as follows:

1 $$\begin{array}{*{20}c} {O_{2}^{*} + {\mathrm{e}}^{ - } \to {\mathrm{O}}_{{2\left( {{\mathrm{sol}}} \right)}}^{ - } } \\ \end{array}$$

2 $$\begin{array}{*{20}c} {{\mathrm{Li}}_{{\left( {{\mathrm{sol}}} \right)}}^{ + } + {\mathrm{O}}_{{2\left( {{\mathrm{sol}}} \right)}}^{ - } \to {\mathrm{LiO}}_{2} } \\ \end{array}$$

3 $$\begin{array}{*{20}c} {2{\mathrm{LiO}}_{{2\left( {{\mathrm{sol}}} \right)}} \to {\mathrm{Li}}_{2} {\mathrm{O}}_{{2\left( {{\mathrm{sol}}} \right)}} + {\mathrm{O}}_{2} } \\ \end{array}$$

4 $$\begin{array}{*{20}c} {{\mathrm{LiO}}_{2}^{*} + {\mathrm{Li}}^{ + } + {\mathrm{e}}^{ - } \to {\mathrm{Li}}_{2} {\mathrm{O}}_{{2\left( {\mathrm{s}} \right)}} } \\ \end{array}$$

Oxygen molecules is firstly reduced on the catalysts surface Eq. (1) and combined with Li^+^ for LiO_2_ Eq. (2). Next, there are two paths from LiO_2_ to Li_2_O_2_ as follows Eqs. (3–4), experiencing the disproportionation involving two LiO_2_ molecules or adsorbed LiO_2_ continuous reduction with an additional Li ion, respectively. The former is the solution-mediated mechanism with large toroidal products, while the latter is the surface growth mechanism with film-like products, influenced by the solvation effect of electrolyte [54]. The fundamental reaction steps offer insights into diverse electrochemical performance, where the product amounts, reaction kinetics and reversibility, are closely correlated with the specific capacity, rate capability, and energy efficiency [14, 17, 59, 60]. Accordingly, recent attention has been paid to exploring efficient cathode catalysts and constructing catalytic mechanisms [14, 27, 61].

Two major issues regarding the discharge products bring challenges to the development of catalysts. The insulation of discharge products Li_2_O_2_ is the first challenge, leading to high recharging voltage and low energy efficiency [27]. Although noble metal catalysts display high electron conductivity and catalytic activity in the cathode of LOBs, the high cost limits their application in large scale [61]. It is an effective way to induce the formation of film-like Li_2_O_2_ products with high electron conductivity and ion transport, which can be decomposed at low voltage [31, 62]. According to Sabatier relations, appropriate adsorption strength and electron transfer are critical for the reversibility of electrochemical reactions [63]. The d-band center is a famous descriptor to evaluate the adsorption strength of reaction species on the catalysts surface, where the center sites can be modulated by alloying [64, 65], heterostructure (such as MoS_2_/NiS_2_ [29]), and single-atom-doped graphene [66]. Moreover, adjusting the electronic structure of catalysts could activate the discharge products by injecting the electron, for example, via d-p orbital hybridization between the catalysts and O_2_ molecules [67], thus promoting the decomposition process. Another challenge stems from the undesirable side reactions, which can be attributed to degradation of carbon materials and electrolyte [17, 59]. In return, the side products accelerate the above passivation process, significantly decreasing the performance of LOBs. It is a good way to build multifunction co-catalysts for the decomposition of Li_2_O_2_ and side products. However, there is limited exploration restricted by the complex reaction products and environment [68]. In Li–CO_2_/O_2_ batteries, some of composite (e.g., Pt/FeNC [69], Ru/NiO [70]) catalyst present highly efficient catalytic properties for the decomposition of LiOH, Li_2_CO_3,_ and Li_2_O_2_. Overall, the slow reaction kinetics are the main reason for the poor performance and parasitic reactions; developing highly efficient cathode catalyst is the broad way to LOBs.

### Critical Role of Anode, Separator, and Electrolyte

In LOBs, lithium anode, separator, and electrolyte each play their own role while being interrelated. Lithium metal draws significant attention on account of its high specific capacity of 3860 mAh g^−1^ and low reduction potential of − 3.04 V versus the standard hydrogen electrode. It endows the battery with remarkable energy storage and output capacity. The separator is responsible for separating the cathode and anode, and providing channel for the transmission of lithium ions. As for the electrolyte, it serves as a medium that allows lithium ions to migrate freely within the battery. Although there are similar components between LOBs and lithium-ion batteries, the commercialized parts in lithium-ion batteries cannot meet the requirements of LOBs. Aspects such as electrolyte design [71], separator modification [49], lithium anode protection [15], and assembly structure design [72] all have a significant impact on the performance of LOBs. In particular, the stability of lithium metal is a critical challenge in all the lithium-based batteries [73]. During the continuous stripping and deposition process of lithium metal, the flat metal surface gradually evolves into dendrites, cracks, and particles, which significantly decrease the battery energy efficiency. In particular, the lithium dendrites can directly penetrate the separator, thus causing short circuit in the battery. Constructing a protective layer with high mechanical strength (such as BN [46] and MXene [45]) is an effective approach to suppress the dendrite. Furthermore, the uniform contact between the liquid and solid enables the electrolyte to possess multiple functions with the assistance of additives, in protecting the anode [74], accelerating the reaction kinetics [75] and stabilizing the chemical environment [71]. However, the semi-open condition in LOBs brings another challenge for the liquid electrolyte resulting from the leakage and volatilization. As mentioned above, the straightforward working processes of the anode, separator, and electrolyte are confronted with serious challenges, and many of these issues have not been satisfactorily resolved yet. Understanding the cooperation of each part and exploring the functionality of 2D materials are of crucial importance for the improvement of battery performance.

## Active 2D Cathode Catalysts for Li–O_2_ Batteries

### Graphene-Based Materials

Graphene and correlated derivatives have been widely studied as the cathode catalysts in LOBs, which can be attributed to its many good qualities [5, 35, 60]. Firstly, the high specific surface area (2630 m^2^ g^−1^) and electronic conductivity allows high density surface to support active sites and insoluble discharge products. Secondly, the excellent mechanical properties and stability of graphene allow it to maintain original structure during various activity modifications. Generally speaking, the pristine graphene with perfect honeycomb is inert for catalytic processes [1]. Hence, most researches are concentrated on the activated graphene with modulation strategies, e.g., edge/defects graphene [23, 76], metal or non-metal-doped graphene [25, 77], and decorated graphene with other catalysts [78].

#### Edge and Defects

The edge boundary of graphene is generally considered as the active sites, which benefit from the dangling groups and valences at corresponding places [19, 24]. Downsizing the materials is one of the efficient methods to produce edges/defects, such as by way of graphene quantum dots. However, graphene dots passivate quickly with products deposition for its small surface area and aggregation tendence [19, 79, 80]. Vertical graphene is a complex thin-film material featuring good characteristics of graphene, such as high electron conductivity, chemical stability, and large surface area. The vertically aligned sheets provide sufficient space for products storage and mass transference [81, 82]. More importantly, it has the highest ratio of edge atoms among any allotrope, giving many active sites [83]. Typically, Su et al. prepared Ru decorated vertical graphene sheets for LOBs, and the large space realized a high capacity of 23,864 mAh g^−1^ and a low charge overpotential of 0.45 V at a current density of 200 mA g^−1^ [84]. Furthermore, Kumar et al. prepared vertical graphene layers via chemical vapor deposition method in which the number of layers can be precisely controlled [34]. The high O_2_ capture capability on the edge site induces the formation of thin flake-like Li_2_O_2_, which can be easily decomposed in the following charging process.

**Fig. 2:**
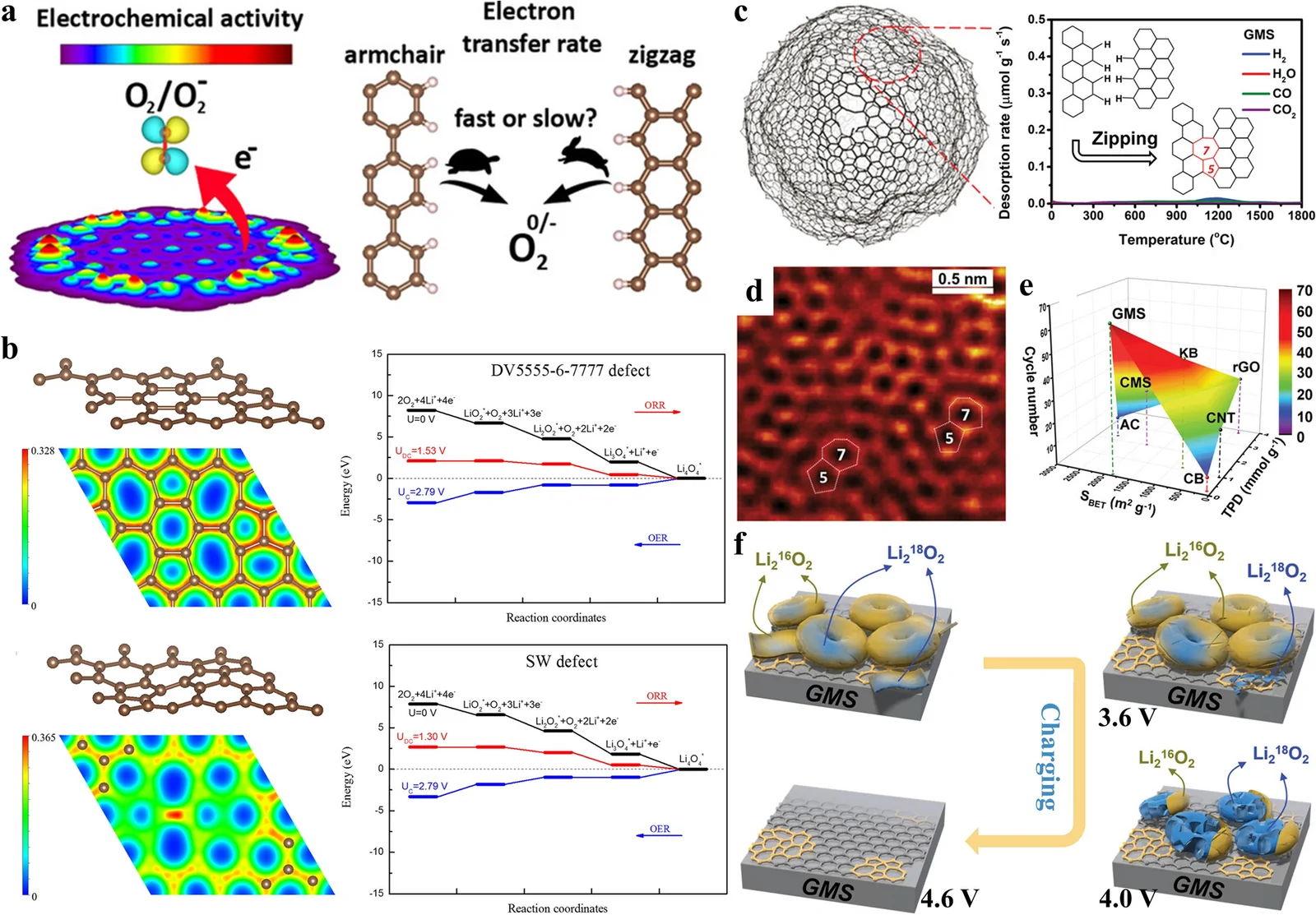
**a** Electrochemical activity mapping of graphene and electro transfer rate on the edge plane [86]. Copyright 2019, American Chemical Society. **b** Electron density counter of defective graphene and corresponding reaction energy diagrams [92]. Copyright 2016, American Chemical Society. **c** GMS model and gas evolution patterns during the temperature programmed desorption measurements. **d** Atomic-resolution TEM image of GMS. **e** Relationship between the physicochemical properties of carbon materials and the cycle performance of LOBs. **f** Charging mechanisms of LOBs for GMS cathode [95]. Copyright 2023, John Wiley and Sons

For graphene edges, two typical edge structures are often observed: along with the zigzag or armchair direction. The former keeps a fraction of active unpaired π electrons, while atoms at armchair edges tend to pair each other, maintaining stable covalent bonds [76, 85]. As a result, the zigzag edge with localized electronic states at the Fermi level displays higher electron transfer and better catalytic activity (Fig. 2a) [86]. A comprehensive calculation conducted by Zheng and co-workers also confirmed these points in LOBs [87]. The zigzag edge as cathode reaction site in LOBs realized an extremely low overpotential of 0.025 V compared to that of the armchair edge about 0.7 V. It is noting that most research of the catalytic difference between zigzag and armchair graphene is theoretical calculations works, which can be attributed to the complex and uncontrollable edge structure in graphene [88, 89]. The catalytic mechanism of different edge types in LOBs can be further explored when overcoming the above problems.

Apart from the boundary edge, the point defects and holes are also active sites in the inside graphene matrix [24]. A point defects are created by removing several carbon atoms, and the remaining atoms are connected to form the pentagonal and heptagonal rings. The rebuild bond increases the electron density and correspondingly improves the catalytic properties [90, 91]. Jiang et al. theoretically evaluated the properties of five defective graphene in LOBs, where DV5555-6–7777 and SW defective graphene exhibit zero-band-gap semiconductor behaviors as well as good stability to electrolyte, resulting in low discharge/charge overpotentials (Fig. 2b) [92]. The holes are one of the large defects, and the inner edge was bound as zigzag or armchair configuration. Besides the high activity of the hole similar to the boundary edge, it also shows additional benefits, such as an increase in surface area and facilitation of mass transport [23, 93]. Zhu et al. applied Ni ions as ignition source to etch carbon atoms and generate controllable hole in graphene layers, forming a tri-continuous path of electrons, ions, and oxygen [93]. The efficient mass transfer and charge transport through the hole accelerate the formation of Li_2_O_2_ on both the outside and inside of the graphene. Therefore, the holey graphene realized a discharge capacity of 7400 mAh g^−1^, 1.7 times increase compared to the original graphene cathode (4300 mAh g^−1^).

Despite the high catalytic properties of carbon atoms near the edge, unfortunately, those atoms tend to contribute to the decomposition of electrolyte molecules or to be attacked by oxygen intermediate and singlet oxygen, forming undesirable side products (e.g., Li_2_CO_3_, HCO_2_Li, and CH_3_CO_2_Li) [14]. Hence, a novel type of graphene mesosponge (GMS) with an extremely small number of edges was synthesized to address this trade-off [94]. Recently, Yu et al. conducted some work about edge-site-free GMS cathode catalysts. (Fig. 2c–f) [95–97]. The graphene with different orientations coalesces each other under high temperatures, building active topological defects in the synthesis process. Density functional theory (DFT) calculation results show that topological defects display high adsorption affinity to LiO_2_ intermediates, leading to the formation of floc-like products that can be completely decomposed under 4.0 V [95]. Meanwhile, the GMS cathode can work for 52 cycles at a high current density of 0.4 mA and large capacity of 0.5 mAh, much better than other types of carbon materials.

#### Dopant Functions of Non-Metal/Metal Atoms

Heteroatom doping is an efficient activity strategy by causing the uneven electron distribution or spin density on neutral graphene matrix, which can be ascribed to the different electronegativities and electronic structure between carbon atoms and the heteroatoms, such as N, B, P, and S, etc. [61, 98]. In particular, the doped–N atoms with high electron-withdraw ability can increase the positive charge density on the adjacent carbon atoms as well as the amount of unsaturated state, increasing electron conductivity of the system and enhancing the ORR performance [82, 85]. In contrast, B atom promotes the electron transfer from Li_2_O_2_ to substrate for its p-type behavior, and thus, B-doped graphene activates the Li–O bond and promote the decomposition of products in the OER process [90, 99]. In light of the role of N and B, Xu group conducts experiment to evaluate the performance of N–B co-doping graphene cathode in LOBs [100]. Furthermore, some of other dual non-metal atom doping carbon systems, for example, N–I [101], N–S [102], and N–P [77, 103] systems, have been studied in LOBs, in which the N atom gives rises to electron conductivity, whereas the second heteroatoms act in increasing active sites, tuning products nucleation and crystallization, prohibiting side products formation, or changing the reaction processes, and therefor exhibit better electrochemical performance as cathode catalysts in LOBs.

**Fig. 3:**
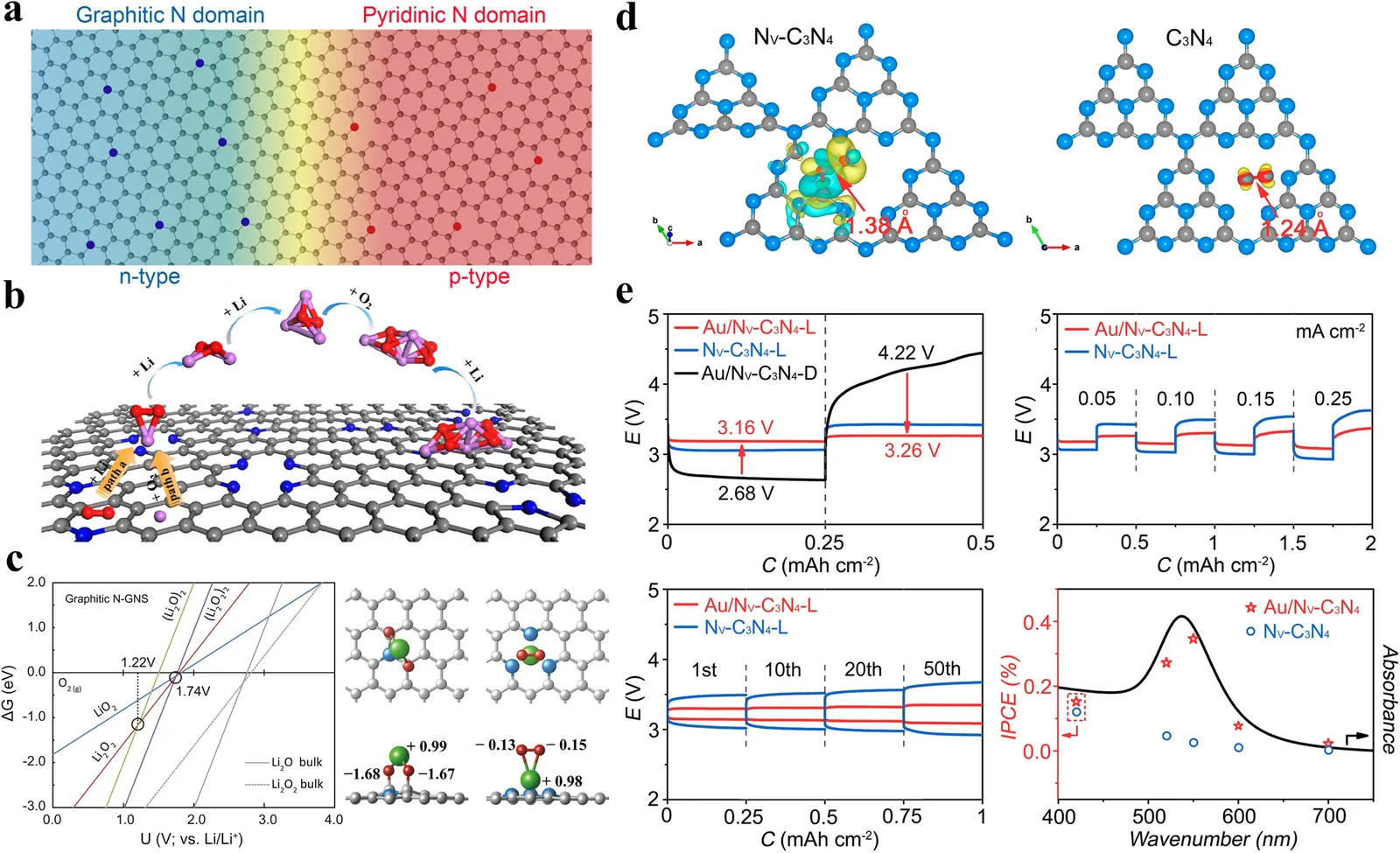
**a** Schematic illustration of the graphitic N and pyridinic N-doped graphene [104]. Copyright 2017, American Chemical Society. **b** Schematics for the reaction mechanism on the N-doped graphene [105]. Copyright 2015, American Chemical Society. **c** Potential dependent Li–O intermediate surface phase diagram on graphitic N substrate. Adsorption configuration of LiO_2_ species is compared on graphitic N (left) and pyridinic N site (right). The negative and positive value indicates the increased and decreased number of electrons [106]. Copyright 2015, Elsevier. **d** Differential charge densities of O_2_-adsorbed N_V_–C_3_N_4_ and C_3_N_4_. **e** Electrochemical performance of Au/N_V_–C_3_N_4_ cathode and plasmonic effect of Au particles from IPCE at different wavelengths [112]. Copyright 2021, National Academy of Sciences

Putting aside the types of heteroatoms, there is still controversy regarding the function of heteroatom coordination structure, such as graphitic N, pyridinic N, and pyrrolic N in the graphene-based matrix. As shown in Fig. 3a, graphitic nitrogen forms three σ bonds with adjacent carbon atoms, while one electron participates in the delocalized π system, rendering nitrogen an electron donor (n-type) and enhancing the electronic conductivity. Pyridinic N contains a lone pair of electrons without participating in π-conjugated system, resulting in p-type behavior [104]. Pyrrolic N is unstable and exhibits weak catalytic properties and has therefore been less studied in applications. Jing et al. calculated results found that pyridinic N exhibits lower overpotential than graphitic N from the reaction diagrams, which favors the nucleation of (Li_2_O_2_)_2_ cluster (Fig. 3b) [105]. However, Yun et al. constructed a potential dependent phase diagram (Fig. 3c) and displayed that the formation of (Li_2_O_2_)_n_ clusters is energetically favorable at graphitic defective site [106]. They also claimed that pyridinic N only facilitates the charge transfer with Li site, which cannot be considered as ORR reaction due to positive total charge transfer in LiO_2_. In contrast, graphitic N plays a pivotal role for LiO_2_ formation by donating electrons to oxygen atoms. For experiments, Wang et al. obtained graphitic N (42.8%) dominates catalysts from soybean-derived carbon under strong KOH etching. The control group etched with KHCO_3_ possess 44.4% proportion of total graphitic N and pyridinic N. The electrochemical results displayed that former exhibits a specific capacity of 9172 mAh g^−1^, about 1.5 times higher than that of the KHCO₃-etched electrode [107]. Li et al. prepared N-doped structure from chitin-derived carbon via H_3_PO_4_ and KOH activation. The H_3_PO_4_ activated materials contain 48.4% graphitic N and 29.8% pyridinic N, whereas KOH etched sample shows 52.6% and 14.6%, respectively. The electrochemical performance indicates that the H_3_PO_4_ activated electrode realizes a capacity of about 8400 mAh g^−1^, which is about 5 times higher than that of the KOH activated materials [108]. It should be emphasized that morphologies differences make it difficult to directly identify N species role in these samples from electrochemical performance. Most of the results present the “collective contributions” for the multiple N-doping species. Ning et al. reported that the electron transfer efficiency follows an “inverse volcano” relationship in pyridinic N and graphitic N, explaining their synergistic catalytic roles [109]. Zhang et al. also emphasized that not all pyridinic N sites are equally active for the ORR, as revealed by their basicity. This variation can be attributed to the fact that pyridinic N is susceptible to the influence of adjacent nitrogen atoms [110]. It can be concluded that complex roles of different N-doped graphene species originate from the different electronic characters, computation methods, and compositional complexity of during electrochemical performance evaluation. Therefore, it is important to investigate the specific roles of different N species in graphene in future studies. A review has summarized the precise synthesis strategies of N-doped graphene [111]. Meanwhile, synergistic effects should be considered in model construction for theoretical calculations.

Another carbon derivative with high N content, g-C_3_N_4_, has been widely applied in catalytic reactions. Wang et al. recently reviewed the literature on g-C_3_N_4_ based cathode catalysts in photo-assisted LOBs [6]. Improving photocatalytic activity is an effective way to improve the performance of the g-C_3_N_4_ cathode in LOBs. For this purpose, Zhu et al. use plasmonic heterojunction of Au particle decorated C_3_N_4_ with N vacancies as a bifunctional catalyst to promote catalytic activities of the visible light-responsive LOBs [112]. The N vacancies accelerate the electron transfer from substrates to adsorbed O_2_, enlarging the O–O bond length (Fig. 3d). Plasmonic metal Au particles were closely attached to the C_3_N_4_ surface, which exhibits a low electron–hole recombination rate, enhanced light harvesting, and wide response wavelength. As a result, the photocathode achieves a high discharge voltage of 3.16 V under illumination, and a low charge voltage 3.26 V with good rate capability and cycle stability (Fig. 3e).

The above strategies mainly modulate and utilize the intrinsic properties of graphene, which could not meet the multifunctional requirements of the cathode in LOBs. At the same time, the identity of the catalytic center remains elusive. To this purpose, the single-atom catalysts (SACs) doped in graphene have gained significant attention for their high catalytic nature, good selectivity, and stability, acting as important and accurate catalytic centers [3, 20, 113]. The atomically dispersed metal usually anchors near the N environment with MN_4_ configuration and promotes electron transfer from metal to adjacent N atom (e.g., PdN_4_ [114], RuN_4_ [115]), which weakens its adsorption strength to reaction intermediates in LOBs. Non-precious metal represents an emerging system with multiple valency states, balanced catalytic activities and costs, allowing for versatile reactant interactions and diverse catalytic mechanisms. For example, the metal in NiN_4_ and CoN_4_ possess positive charge from 0 to + 2, which can modulate the crystallization of Li_2_O_2_ products [116, 117]. Zheng et al. compared the performance of CoN_4_ and CoN_3_ coordination, and near-free CoN_3_ tends to form the O–Co–N_2_ structure during the ORR process. This evolution conductive to the surface adsorption and activation of key oxygenated intermediates for efficient ORR [118]. In addition, the near-free Co has a better lattice match with the (100) plane of Li_2_O_2_, forming oriented sheet-like Li_2_O_2_. In LOBs, CoN_3_ presents high capability to decompose the products with low charging overpotential of 0.51 V even after 3500 h.

**Fig. 4:**
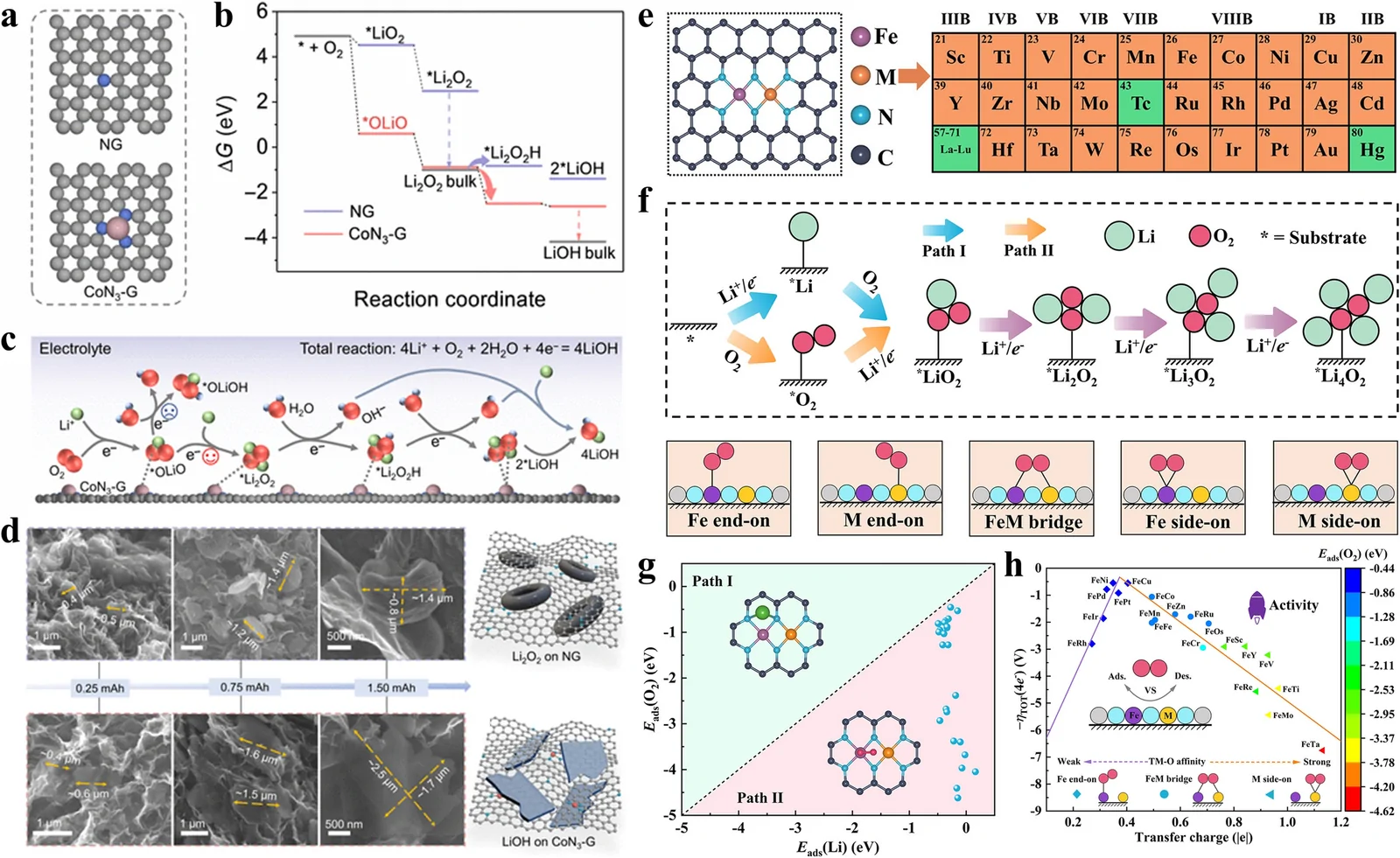
**a** Atomic structures of NG and CoN_3_-G. **b** Free energy diagram of ORR for CoN_3_-G and NC.** c** Illustration of electrochemical reaction mediated by CoN_3_-G in LOB system. **d** Ex situ SEM images for the observation of discharge products on NG and CoN_3_-G cathode at different discharge states [57]. Copyright 2024, Chinese Chemical Society. **e** Structural prototype of FeM@NC and 27 transition metals candidate. **f** Schematic diagram of reaction pathways for the ORR process and five proposed O_2_ adsorption modes on the FeM@NC.** g** Adsorption energy of O_2_ and Li elucidates the nucleation pathway of LiO_2_ on FeM@NC following Path II. **h** Volcano plot for total overpotential versus charge transfer of O_2_ [66]. Copyright 2024, American Chemical Society

Recently, some studies have confirmed that the SACs play a role in tuning reaction routes from the 2e^−^ pathway of Li_2_O_2_ to the desired 4e^−^ pathway of LiOH under a moisture environment, where the LiOH products can be easily decomposed at low charging voltage, further improving the electrochemical performance of LOBs. For instance, Zhang et al. reported that CoN_4_ acts as “water-trapping” catalyst and induces the transformation of LiO_2_ to LiOH [119]. Furthermore, Huang et al. calculated that the LiOH displayed lower thermodynamic free energy than Li_2_O_2_ on the CoN_3_ SACs surface (Fig. 4a, b) [57]. The experimental results displayed that the LiOH is directly formed, which can be ascribed to the cleavage of O–O bond on the CoN_3_ site (Fig. 4c, d). In consequence, the direct pathway with 4e^−^ avoids the formation of side products and maintains the good electrochemical performance of LOBs.

On the basis of SACs, introducing another active single atom is an effective way to form diatomic center, which boosts both ORR and OER performance. Currently, the research about dual-atomic catalysts (DACs) in LOBs is primary stage due to the challenges posed by increased selection and complexity of possible binary element combinations. Lim et al. proposed that the loading sequences between Ni and Fe atoms in DACs affect the electrochemical performance of LOBs [120]. They found that the loading order affects the metal yield, and the first loading of Fe atoms leads to the increase in metal yield. Finally, the NiFe DACs with Fe first loading achieve a long cycle life over 200 cycles with limited capacity of 1000 mAh g^−1^. Furthermore, Fu et al. synthesized dual Co single-atom catalysts with precise pair spacing configuration at angstrom scale [121]. This special configuration increases the interaction between substrate and reaction intermediates and also distorts the structural alignments of products, thereby facilitating delithiation and oxidation in the charging process. Theoretical calculations provide an efficient method for rapidly screening and rationally designing advanced electrocatalysts for LOBs [66]. In a recent study conducted by Mao and co-workers [66], 3*d*, 4*d*, and 5*d* transition atoms are selected as dopant metal M for FeN_4_ with total of 27 types of FeM dimers (Fig. 4e). Two reaction pathways are considered for the initial ORR process, based on the favorable adsorption of Li or O_2_, as well as the formation of different products of Li_2_O_2_ and Li_4_O_2_ (Fig. 4f, g). Ultimately, the FeNi and FeCu dimers stand out with the lowest total overpotential of 0.54 and 0.55 V (Fig. 4h), demonstrating significant potential for the high-performance cathode catalysts of LOBs.

#### Catalysts Decoration

**Fig. 5:**
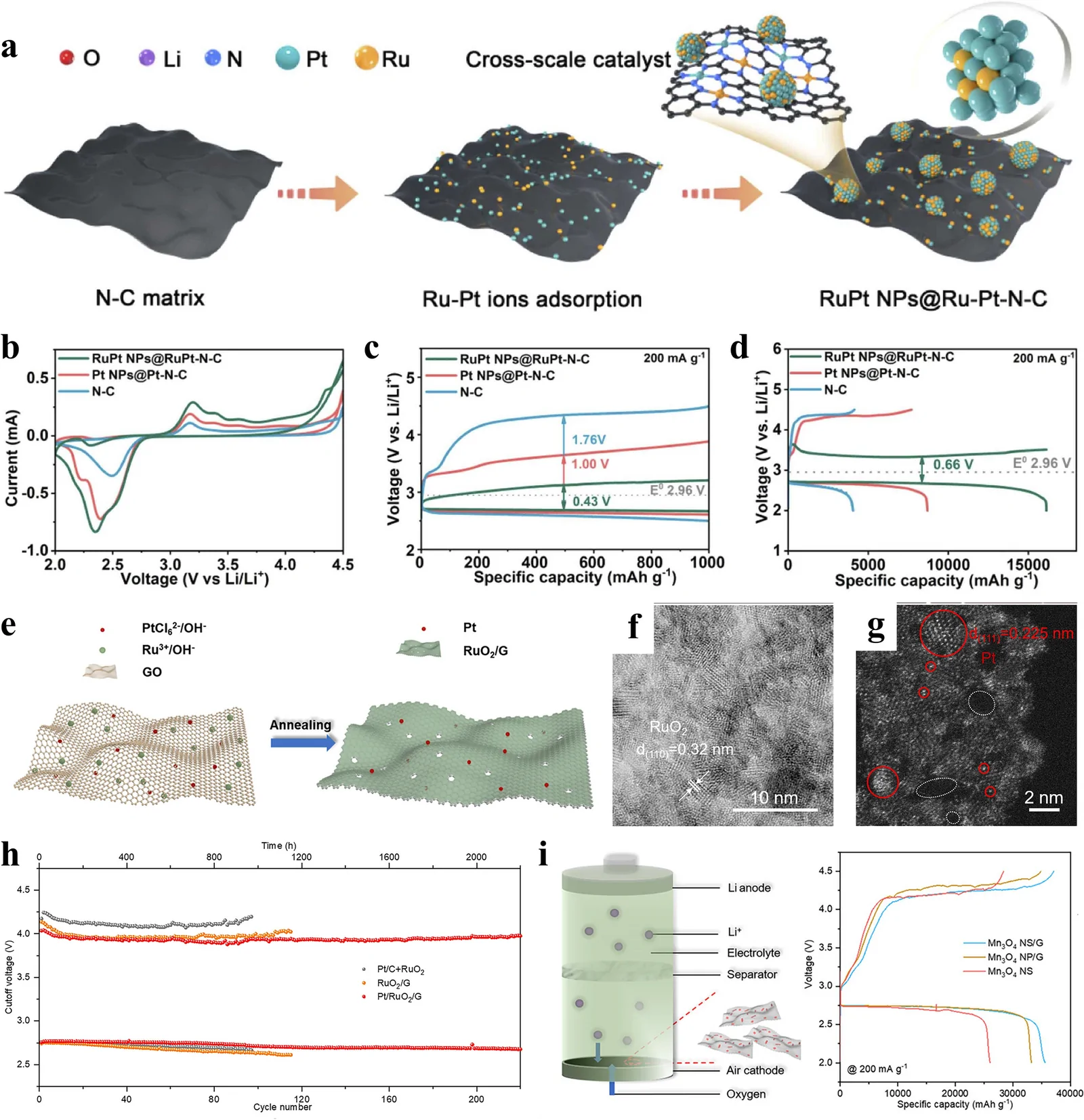
**a** Schematic illustration of the synthetic process for RuPt-loaded graphene. **b** CV curves of different electrodes. Electrochemical curves of the different electrodes **c** with limited capacity of 1000 mAh g^−1^ and **d** full discharge/charge capacity at current density of 200 mA g^−1^ [25]. Copyright 2024, Elsevier. **e** Scheme of the fabrication procedure of Pt/RuO_2_/G. **f** TEM and **g** HADDF-STEM images of Pt/RuO_2_/G. **h** Cyclability of Pt/RuO_2_/G cathode at 200 mA g^−1^ under a cutoff capacity of 1000 mAh g^−1^ [78]. Copyright 2023, Elsevier. **i** Electrocatalytic performance of Mn_3_O_4_/graphene cathode catalysts in LOBs [125]. Copyright 2022, American Chemical Society

A layer of highly active catalysts decorated on the graphene surface can shift the catalytic center and protect the carbon surface, enhancing the electrochemical performance of LOBs [27, 98, 103]. In this circumstance, the decoration catalysts act as active sites with high ORR/OER performance, while the graphene supports a large amount of discharge products. Li et al. demonstrate that CoRu nanoparticles could effectively suppress side reactions caused by exposed carbon sites [122]. Palani et al. designed well-distributed GrZnCo_3_ particles on graphene, and the catalysts enable LOBs to achieve high discharge capacity of 13,500 mAh g^−1^ and long cycle life over 400 cycles at 100 mA g^−1^ with limited capacity of 500 mAh g^−1^ [123]. The active sites on the functional graphene can also be enhanced by extra decorations [25, 124]. Guo et al. reported a cross-scale catalytic mechanism in the RuPt-loaded graphene catalysts (Fig. 5a) [25]. Pt nanocrystals are fully activated by Ru atoms, which promote the ORR kinetics for the formation of easily decomposed nanoflower Li_2_O_2_, realizing improved electrochemical performance with low overpotentials about 0.43 V at a current density of 200 mA g^−1^ (Fig. 5b–d). Li et al. demonstrate durable bifunctional electrocatalysts by coupling advanced ORR catalysts Pt single atom with effective OER catalyst RuO_2_, strongly anchored on graphene (Fig. 5e) [78]. The combined merits come from efficient spatial confinement by integrating Pt cluster into ultra-small RuO_2_ nanoparticle, exerting improved electrochemical performance (Fig. 5f–h). In addition, graphene can avoid agglomeration of catalysts in the synthesis process, increasing the capacity of LOBs. The Mn_3_O_4_ nanosheets are uniformly deposited on the graphene surface and applied as cathode catalysts (Fig. 5i) [125]. Compared with the pure Mn_3_O_4_ cathode, the Mn_3_O_4_/graphene cathode significantly increases the discharge capacity to 35,583 mAh g^−1^ at current density of 200 mA g^−1^ with low-voltage working window.

#### Correlation among a Different Activation Engineering and Electrochemical Performance in Graphene

Graphene provides an ideal platform to probe the correlation between different activation engineering and catalytic mechanisms. The different activation engineering accompanies varying degrees of electronic structure modulation and adsorption behavior and enhances the catalytic activity, which in turn guides the next design and development: (1) Balance between activity and stability. Pore defects and edge boundary exploit the properties of graphene, in which the modulated electronic structure is the catalytic initiator for the ORR process. However, the low stability of activated atoms leads to side reactions during working process, gradually decreasing the electrochemical performance of LOBs. Non-metal doping provides an activated point in the graphene without compromising structural stability. (2) Precise design and synthesis. Although the multitude of elements offers various potential choices for the graphene, the complex coordination structures obscure the catalytic mechanisms, resulting from the challenges of precisely controlling dopant structure, quantity, and locations. Hence, studies on simple and controllable synthesis processes for functional graphene are essential. This is a crucial step, not only to utilize the catalytic activity of graphene as much as possible but also to enhance its effectiveness as a substrate for other catalytic components. (3) Synergic function. The metal single atoms doping or catalyst decoration significantly regulates the electronic structure of graphene at a large scale, which greatly enhances the catalytic performance. The morphology design of graphene also affects the loading of the active ingredient as well as the deposition of products. Overall, rational activation engineering is necessary to maximize the performance of graphene materials, enabling their realistic application in LOBs.

### Transition Metal Oxide and Hydroxides

Transition metal oxides (TMOs) and hydroxides (TMHs) have been applied for kinds of electrocatalytic processes owing to their many advantages, including efficiently catalytic activity, multiple valences, good stability, and low cost [7, 8]. In contrast to bulk phases, 2D TMOs and TMHs are becoming more promising candidates with enhanced conductivity, large exposed area, and activated metal sites. Meanwhile, it is easy to modulate these materials through activation engineering such as vacancies [126], doping [37], heterojunctions [127], and therefore boosting their catalytic performance in cathode of LOBs.

#### Transition Metal Oxides

**Fig. 6:**
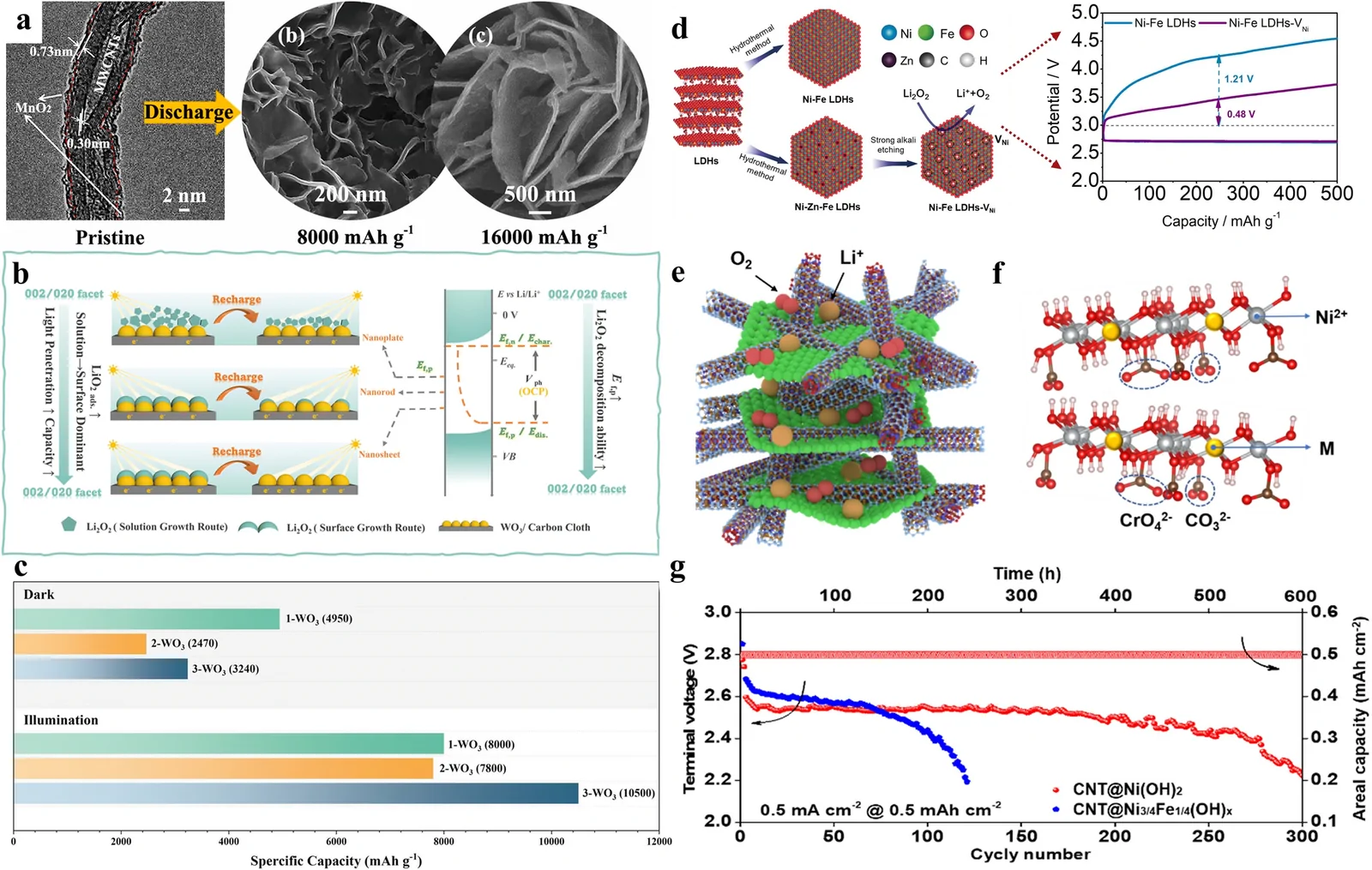
**a** Morphological evolution of discharge products on surface of δ–MnO_2_ wrapped CNT [135]. Copyright 2019, American Chemical Society. **b** Schematic of the facet-controlled Li_2_O_2_ growth routes and WO_3_ photocatalytic activity in photo-assisted LOBs. **c** Galvanostatic discharge profiles of the three photocathodes at 50 mA g^−1^ (0.02 mA cm^−2^) with and without illumination [31]. Copyright 2023, Royal Society of Chemistry. **d** Schematic illustration for preparation of Ni–Fe LDHs with Ni vacancies [151]. Copyright 2021, Elsevier. **e** Schematic illustration of cathode reaction on Ni-based LDHs with conformal contacts to CNTs. **f** Substitute CrO_4_^2−^ for CO_3_^2−^ and replace part of Ni^2+^ with M = Fe^3+^, V^3+^, and Co^2+^. **g** Cycling stability test with an area capacity limit of 0.5 mAh cm^−2^ at 0.5 mA cm^−2^ [156]. Copyright 2022, Elsevier

There exist two main classes of 2D TMOs in terms of crystal structure: layered TMOs and non-layered TMOs [7, 128]. The former with a graphene-like structure consists of a few materials such as MoO_3_, WO_3_, and V_2_O_5_, in which the layers are connected by van der Waals forces. Instead, the non-layered TMOs with typical 3D structures occupy a large group and stand out for that with ultrathin morphologies. However, the main challenge for preparing 2D non-layered TMOs comes from the strong chemical bond in the structure. Accordingly, a simple and general strategy is applicable to prepare various TMOs that use water-soluble salt as growth templates, with the thickness of below 2 nm [129]. It is a better way to prepare thin TMOs by converting thin precursor into corresponding oxides [32, 130–132]. Taking Mn-based oxides as an example, Mn_2_O_3_ thin nanosheets are synthesized by inducing phase transition of layered MnO_2_ under thermal conditions [133]. The heat treatment led to the formation of holes in the surface, which exposes more active metal sites. In LOBs, the holey Mn_2_O_3_ cathode displayed better performance than bulk Mn_2_O_3_, especially in enhancing the Coulombic efficiency. Furthermore, other strategies, such as facet modulation [125, 134], composition [135, 136], and heterojunction [67, 137], have also been shown to improve the catalytic performance of Mn-based TMOs in the LOBs. Ma et al. reported a composite with δ–MnO_2_ wrapped on the surface of multiwall carbon nanotubes (CNT) in the form of a monomolecular layer through the chemical binding (Fig. 6a) [135]. The dominant (002) facet and abundant oxygen vacancies of the δ–MnO_2_ increase the adsorption of O_2_ and activate the O–O bond, promoting the ORR process.

Among 2D layered TMOs, MoO_3_ consists of distorted [MoO_6_] octahedral layers in an orthorhombic structure, where the octahedra are corner-sharing along the a and z axes and edge-sharing along the b axis. Introducing oxygen vacancies in MoO_3_ can create negative-charge surface to provide strong adsorption to active oxygen and at the same time, the exposed metal can serve as an efficient site for decomposition of products [138]. Nevertheless, as the deposit of insulation Li_2_O_2_, the low conductivity of MoO_3_ restricts its catalytic performance. It has been reported that composite with CNT or building heterojunctions in MoO_3_ will promote electron transfer and improve the electrochemical performance in LOBs [139, 140]. Sun et al. studied the MoO_x_@Ti_3_C_2_ MXene catalysts by in situ incorporation of interfacial oxygen bridge binding with Mo–O–Ti units, optimizing the interface electronic structure and improving the conductivity. Finally, the MoO_x_@Ti_3_C_2_ MXene cathode delivers a low overpotential of 0.75 V and a stable working state over 300 cycles at a high current density of 2500 mA g^−1^ [139]. Furthermore, the 2D TMOs have also been studied as photocatalysts in LOBs due to the typical semiconductor characteristics. Recently, MoO_3_@Fe_2_O_3_ [141] and WO_3_@TiO_2_ [142] heterostructures are prepared to boost the photocatalysis performance in LOBs. Moreover, the oxygen vacancies in MoO_3_ have been demonstrated to enhance light-harvesting capability [138, 143]. Meanwhile, WO_3_ has also been studied in photocatalytic Li–O_2_ systems owing to its good stability and narrow band gap. Chen group investigated the facet-engineering and active sites relationships for WO_3_ photocatalysts in LOBs [31, 144]. The results confirm that the controllable pathways of Li_2_O_2_ formation can be realized from solution growth mechanism to surface growth mechanism by increasing exposed (002)/(020) facet ratio (Fig. 6b). These can be attributed to the high adsorption strength of (002) facet to oxygenated intermediates. The WO_3_ can distort the structure of Li_2_O_2_ with Z-type heterojunction under light irradiation, which favors the further formation of Li_2_O_2_ and delivers a high capacity of 10,500 mAh g^−1^, three times higher than that of the cathode without illumination (Fig. 6c).

#### Transition Metal Hydroxides

For typical TMHs, layered double hydroxides (LDHs) consist of brucite-like layers containing multiple positively charged cations, along with an interlayer region that compensates with charge-balancing anions and solvation molecules. These characteristics allow LDHs to receive extensive attention for their favorable properties, such as tunable surface chemical properties, abundant host metal ions, and interlayer anions, which enable them to exhibit efficient performance in processes like water splitting [145], ORR/OER [146], and other electrocatalytic reactions [147, 148]. However, LDHs still suffer from low conductivity, which is a significant obstacle to their further development. Therefore, the carbon matrix CNT and graphene are introduced to composite with LDHs [81, 149, 150]. While the LDHs act as the catalytic center, and the carbon matrix provides a conductive network that facilitates the rate capability. Moreover, LDHs exhibit great flexibility in terms of composition, electronic states as well as intrinsic properties. For example, Zhou et al. implanted cationic Ni vacancies in NiFe-LDHs, which accelerated the rate-limitation step for LiO_2_ oxidation, resulting in low charge overpotential of 0.48 V at 0.1 A g^−1^ with capacity cutoff of 500 mAh g^−1^ (Fig. 6d) [151]. In another work, anionic oxygen vacancies are applied to form the unsaturated metal ions in NiCo-LDHs, which greatly enhance electrochemical performance for the ORR process [152].

The large interlayer space is a great treasure for LDHs in modulating a wide range of properties, such as electronic regulation, avoiding aggregation, and maintaining functional moleculars [153–155]. Typically, Lu et al. intercalate negatively charged RuO_2.1_ into the layers of CoFeNi LDHs, which serve as efficient carbon-free cathode catalysts in LOBs [154]. The structure results in strong interfacial electronic coupling and better electrical conductivity, thereby accelerating catalytic performance. Zhao et al. adopt CrO_4_^2−^ assisted in situ growth method to obtain thin flexible Ni-based LDHs with conformal contacts to CNTs (Fig. 6e, f), and the micro-morphology can be tailored by another coupling metal ions (e.g., Fe, Co, V) [156]. Finally, the optimized NiFe-LDHs cathode delivers a long working life of 300 cycles with a limited capacity of 0.5 mAh cm^−2^. Considering the inevitable function of redox mediators (RMs) in LOBs, the layer space can serve as catalytic space for active RM molecule and avoid side reactions due to the shuttle effect [153]. Recently, high-entropy MnFeCoNiCu LDHs have also been studied in water splitting and demonstrate high OER activity and promising stability, further widening the choice of LDH categories [145]. As discussed above, we believe that the easily adjustable LDHs will show great potential as cathode catalysts for LOBs in future studies.

### MXene-Based Catalysts

MXenes, one of new 2D materials, have been extensively studied due to their high metallic conductivity, large surface area, tunable layer structure, and surface chemistry, making them highly desirable for electrocatalytic conversion reactions [9, 157–159]. Until now, MXene family has more than 30 stoichiometric compositions that have been experimentally synthesized and most are theoretically determined. Zheng et al. reviewed the application of MXene materials in LOBs, which listed the performance of MXene with different functional groups, defects, and other decorations [10]. In this section, we concentrate on the different catalytic sites of MXene materials reported in recent studies, including the sources, differences, and catalytic mechanisms.

#### Active Sites from MXene Compositions

**Fig. 7:**
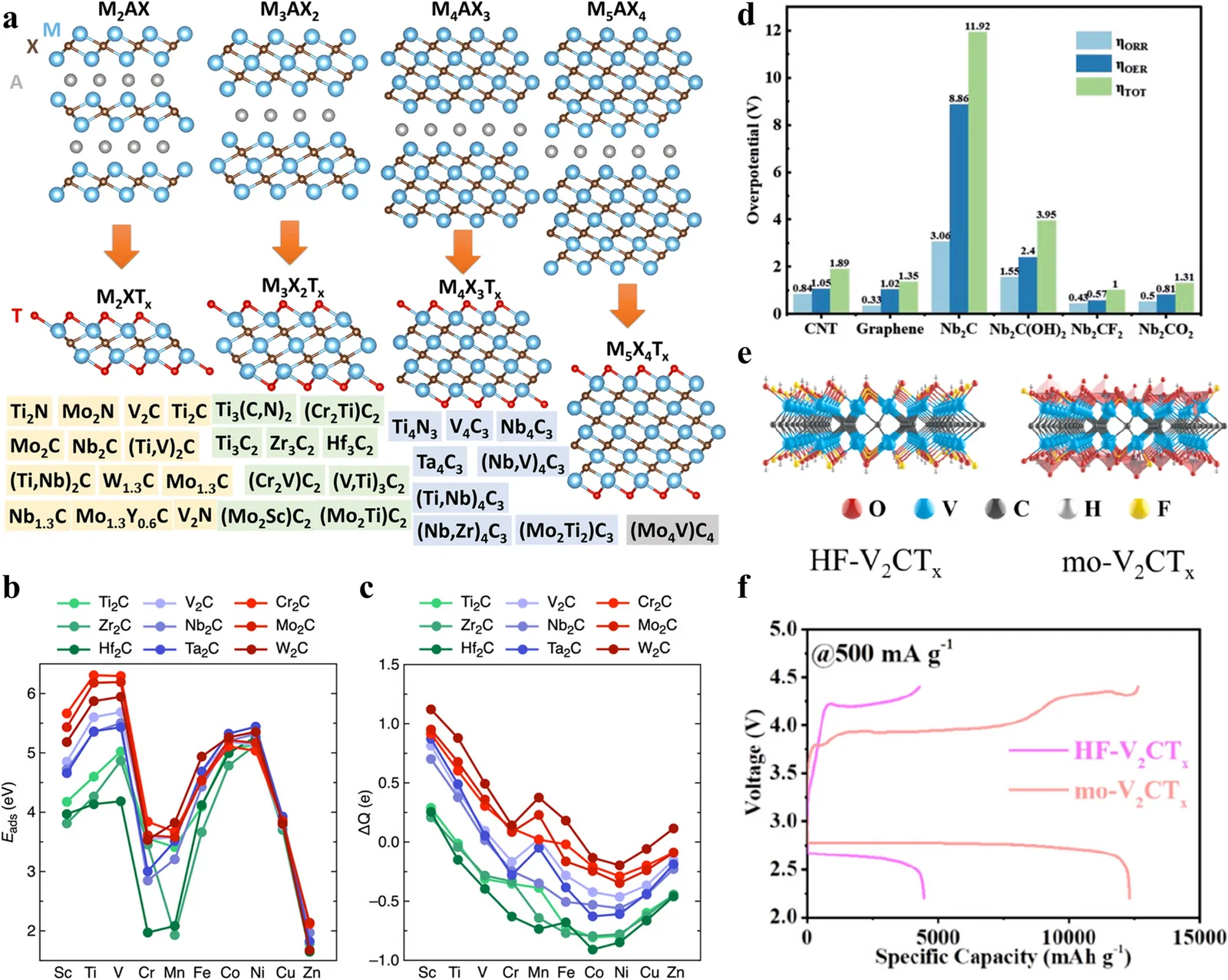
**a** Schematic illustration of the structures of M_2_XT_x_, M_3_X_2_T_x_, M_4_X_3_T_x_, and M_5_X_4_T_x_ from the structures of corresponding MAX phases [9]. Copyright 2023, Royal Society of Chemistry. **b** Adsorption energy of 3d adatoms on the MC_2_ MXene surface. **c** Bader analysis of the adatom charges (ΔQ) anchored on MC_2_ MXenes surface [163]. Copyright 2022, American Chemical Society. **d** Overpotential comparison including ORR, OER, and total overpotentials for Nb_2_C MXene, graphene, and CNT [30]. Copyright 2021, John Wiley and Sons. **e** Structure of mildly oxidized V_2_CT_x_ MXene, and **f** initial full discharge/charge capacity [162]. Copyright 2021, American Chemical Society

MXene materials are synthesized by selectively etching the “A” element from the parent MAX phases, resulting in the general formula of M_n+1_X_n_T_x_ (Fig. 7a) [9]. Here, M, X, and T_x_ represent d-block transition metals, N and/or C atoms, and terminal surface groups (such as –F, –O, and –OH), respectively. Currently, various pristine MXenes, such as Ti_3_C_2_ [160], Ti_2_C [161], Nb_2_C [30], and V_2_C [162], have verified the electrochemical performance of the cathode in LOBs. A theoretical calculation was conducted to evaluate the properties of nine different M_2_C MXenes when anchored with a series of metal atoms (Fig. 7b, c) [163]. There are both similarities and differences among the various MXene candidates. Firstly, the adsorption process for all adatom@M_2_C systems is exothermic, with the hcp and fcc sites being the most often preferred regardless of the types of both adatoms and MXenes. Secondly, it is also noticed that the adatoms follow similar trends on all MXenes but with marked differences, which are noticeably large for the early 3d metals and decrease when moving along the series. These can be attributed to the different electronic configurations of “M” in the *d*^2^, *d*^3^, and *d*^4^-M_2_C systems. Therefore, altering the types of “M” in MXenes can effectively regulate their intrinsic properties and catalytic capability for electrochemical reactions. In addition, theoretical studies show that the number of layers has an effect on the catalytic performance [164, 165]. Increasing the number of layers will improve the electronic conductivity and shift down the d-band center of the materials, which can decrease the adsorption strength of reaction intermediates and promote the OER process. For Ti_n+1_C_n_, the calculated catalytic capability follows a trend: Ti_2_C < Ti_3_C_2_ < Ti_4_C_3_ < Ti_4_C_3_O_2_ < Ti_3_C_2_O_2_ < Ti_2_CO_2_ [164]. Indeed, electrochemical performance confirms that Ti_3_C_2_ catalysts exhibit favorable long-period stability for about 240 cycles with a high limited capacity of 1000 mAh g^−1^, while the Ti_2_C only works for 116 cycles at a 600 mAh g^−1^ capacity [160, 161].

Moreover, there are usually functionalized terminal groups inevitably on the MXene surfaces, which are correlated with synthesis methods and thermal treatment in terms of group types and termination stability. The properties of functionalized groups are identified as playing a crucial role in ORR and OER processes by regulating the electronic conductivity, ionic transference rate, and adsorption strength [10]. For instance, –OH terminations on MXene inhibit the I_3_^−^ migration, preventing the detrimental shuttle effect of RM species in LOBs [166]. Li et al. calculated the adsorption strength and plotted the reaction diagrams for the Nb_2_C surface with different terminations (Fig. 7d) [30]. The –F terminations exhibit low charge transference after adsorbing LiO_2_, suggesting that the catalytic sites will quickly become saturated and degrade the catalytic activity. In contrast, Nb_2_C with –O terminations shows improved electrochemical performance due to its appropriate affinity to reaction species, delivering about 65 cycling life with a limited capacity of 1000 mAh g^−1^ under a high current density of 3 A g^−1^. Luckily, the –O terminations are preferred on the MXene surface regardless of thermal stability and synthesis conditions. Jiang and co-workers investigated a series of mildly oxidized MXenes (Nb_2_C, Ti_3_C_2_, and V_2_C) as cathode catalysts in LOBs, in which V_2_C delivers the smallest onset OER potential and a large response current density (Fig. 7e) [162]. Meanwhile, the mo–V_2_CT_x_ contains remarkably fewer F groups compared to HF–V_2_CT_x_, contributing to a high capacity of 12,304 mAh g^−1^ at current density of 500 mA g^−1^ (Fig. 7f). This indicates that the formation of –O terminations create more catalytic sites and expedites the reaction kinetics of ORR/OER process, resulting in high-performance LOBs. In conclusion, besides the common MXenes, it is still urgent to synthesize more novel MXene materials based on the calculated predictions and impose appropriate functional groups to further enhance their catalytic properties.

#### Active Sites from Incorporated Species

**Fig. 8:**
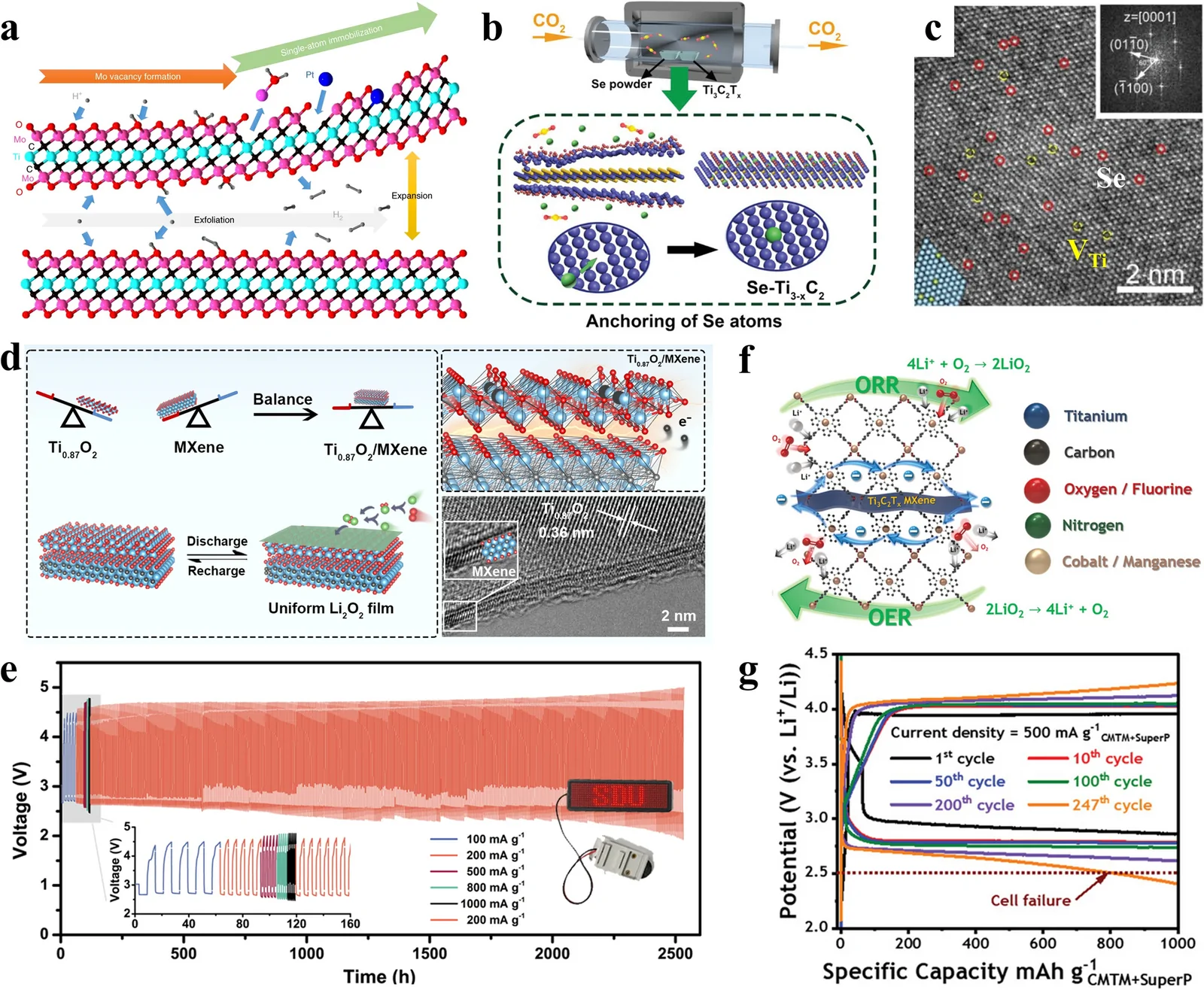
**a** Illustration of the synthesis mechanism for Mo_2_TiC_2_O_2_ with Pt single atoms [169]. Copyright 2018, Springer Nature. **b** Schematic illustration of the synthesis process and **c** HADDF-STEM image of the Se doped Ti_3_C_2_ catalyst [170]. Copyright 2021, John Wiley and Sons. **d** Electrocatalytic mechanism of Ti_0.87_O_2_/MXene catalysts in LOBs. **e** Rate performance of Ti_0.87_O_2_/MXene electrode with a fixed capacity of 600 mAh g^−1^ [173]. Copyright 2024, John Wiley and Sons. **f** Working principle and **g** electrochemical performance of cobalt-manganese organic framework-MXene bifunctional electrocatalyst with an electron hopping mechanism in LOBs [36]. Copyright 2023, John Wiley and Sons

Like graphene, MXenes have also been widely studied as the substrate for extra active atoms and particulate catalysts, utilizing their characteristics of large surface area, good conductivity, and efficient mass transfer. One of the breakthroughs for MXene substrates is overcoming the single composition of graphene, as discussed in Sect. 3.3.1. On the one hand, MXenes with various types provide multiple dimensions and degrees of regulation in electronic structure, adsorption strength, and catalytic performance when incorporated with other species. On the other hand, introducing atomic dopants into MXenes, such as N, P, and S, can generate extra catalytic sites on separation layers, modulate the d orbital electronic states, and adjust the interaction behaviors. Chen’s group reviewed the MXene-based single-atom catalysts for energy conversion applications [158]. For instance, the N dopant in Ti_3_C_2_ MXene effectively modulates the 3*d* orbital occupation of Ti and accelerates the electron exchange between Ti 3*d* and O 2*p* orbitals, improving the ORR performance [167]. In addition, the N content is closely related to the conductivity of MXene where the proper substitution of C with N atoms can effectively improve the conductivity [168]. Among single metal atoms (Mn, Fe, Co, Ni, Cu, Zn, etc.), the Cu-anchored Ti_2_NO_2_ MXene shows the best OER performance by increasing the electronic states at the Fermi level and maintaining stable interactions on the surface without aggregation [157]. Precious Pt atoms doped into the Mo vacancies of Mo_2_TiC_2_ MXene, in which the positively charged Pt atoms and MXene contribute to the exceptional hydrogen evolution performance and stability (Fig. 8a) [169]. The exposed Mo atoms also catalyze hydrogen reduction, and the produced hydrogen further expands the layer structure, resulting in more active sites. In LOBs, the semi-metallic Se atoms are doped into the Ti sites of Ti_3_C_2_ MXene as catalytic centers, accompanied by the electron transfer from C atoms to Se atoms through Se–C bonds (Fig. 8b, c) [170]. During the ORR process, the Se-involved moieties possess high adsorption strength for LiO_2_ intermediates, endowing full interface contact between the active sites and Li_2_O_2_ nanoarrays. Furthermore, the large surface area of MXene provides a uniform deposition substrate for catalytic particles. Pt nanoparticles with low loading content are embedded into the surface or layer spaces of MXene, where the Pt particles are chemically adsorbed by Ti atoms [171]. The abundant pores and active sites in Pt–Ti_3_C_2_ also facilitate oxygen diffusion and the accumulation/decomposition of Li_2_O_2_ during the cycling process. Therefore, it is vital to rationally design the type and structure of incorporated species on the MXene to maximize the catalytic properties.

#### Dual Active Sites from Both MXene and Incorporated Species

For the reversible catalytic processes with multiple reaction steps, it has been established that the electrocatalytic performance has a close correlation in bifunctional catalytic capability and proper adsorption strength (neither too strong nor too weak) of reaction species. Constructing the face-to-face heterostructure will greatly exert this modulation strategy for MXene cathodes in LOBs [139, 172–174]. In particular, the Ti_0.87_O_2_ and Ti_3_C_2_ MXene monolayers are applied to construct 2D heterostructure, which balanced the adsorption strength to the reaction species (Fig. 8d) [173]. The heterostructure provides abundant active sites and electronic compensation through the built-in electronic field, endowing the rapid reaction kinetics and long cycling life spans (Fig. 8e). Recently, the Ti_3_C_2_ MXene and Mo_4/3_B_2-x_ MBene superlattice is fabricated, and large work function difference between each other powers the charge transfer at the interface from Ti to Mo site [175]. This decreases the d-band center of Mo atoms, thus optimizing the adsorption of intermediate product LiO_2_. In situ surface oxidation is another simple and direct methods to modulate the catalytic performance for MXene materials [176]. In contrast, in order to avoid the overoxidation of MXene to metal oxide, Nam et al. reported bimetallic cobalt-manganese organic framework-Ti_3_C_2_T_x_ MXene with strong oxidation resistance [36]. The formed metalloporphyrin structure and unpaired electrons between cobalt–manganese organic framework and Ti_3_C_2_T_x_ MXene improve the electrocatalytic activity, durability, and electrical conductivity through electron hopping mechanism (Fig. 8f, g). Moreover, carbon decorations are one of popular strategies by increasing the system electron conductivity, ion transport rates, and final catalytic performance [177, 178]. The rich research, especially in theoretical calculations, demonstrated that the MXene catalysts play a key role in the application of LOBs. However, there are still some issues that need to be addressed and elucidated, such as the selection between oxidation for building heterostructure and the surface protection for stability. All in all, with the exploration of novel synthesis and modulations, it is confident that MXene materials will achieve more progress in electrocatalytic reactions.

### 2D Transition Metal Dichalcogenides

Recently, 2D TMDs have received broad research interests, owing to their good catalytic activity features and higher conductivity than oxides [37, 179]. TMDs are represented in MX_2_ stoichiometry, where M is transition metal belonging to groups IVB-VIIIB and X is a chalcogen from group VIA. For the crystal structure, take MoS_2_ as instance, the stacked units consisted of two layers of S and one metal Mo layer in unique S–Mo–S structure. Besides, contingent on the d electron configuration, TMDs manifest different coordination patterns within the stacked layers, e.g., 1T, 2H and 3R phases, resulting in the different catalytic characters [180]. In addition, selenide-based TMDs typically exhibit higher electrical conductivity than sulfide analogues owning to their larger atomic radius and enhanced electronic delocalization. Here, this section first elucidated the special catalytic behavior between the uniform surface and edge plane. Meanwhile, the recent advancements of 2D metal sulfides and selenides as high-performance cathode catalysts are reviewed, including the electronic modulation from point sites to surface, and structural designment.

#### Catalytic Anisotropy between the Surface and Edge Facets

**Fig. 9:**
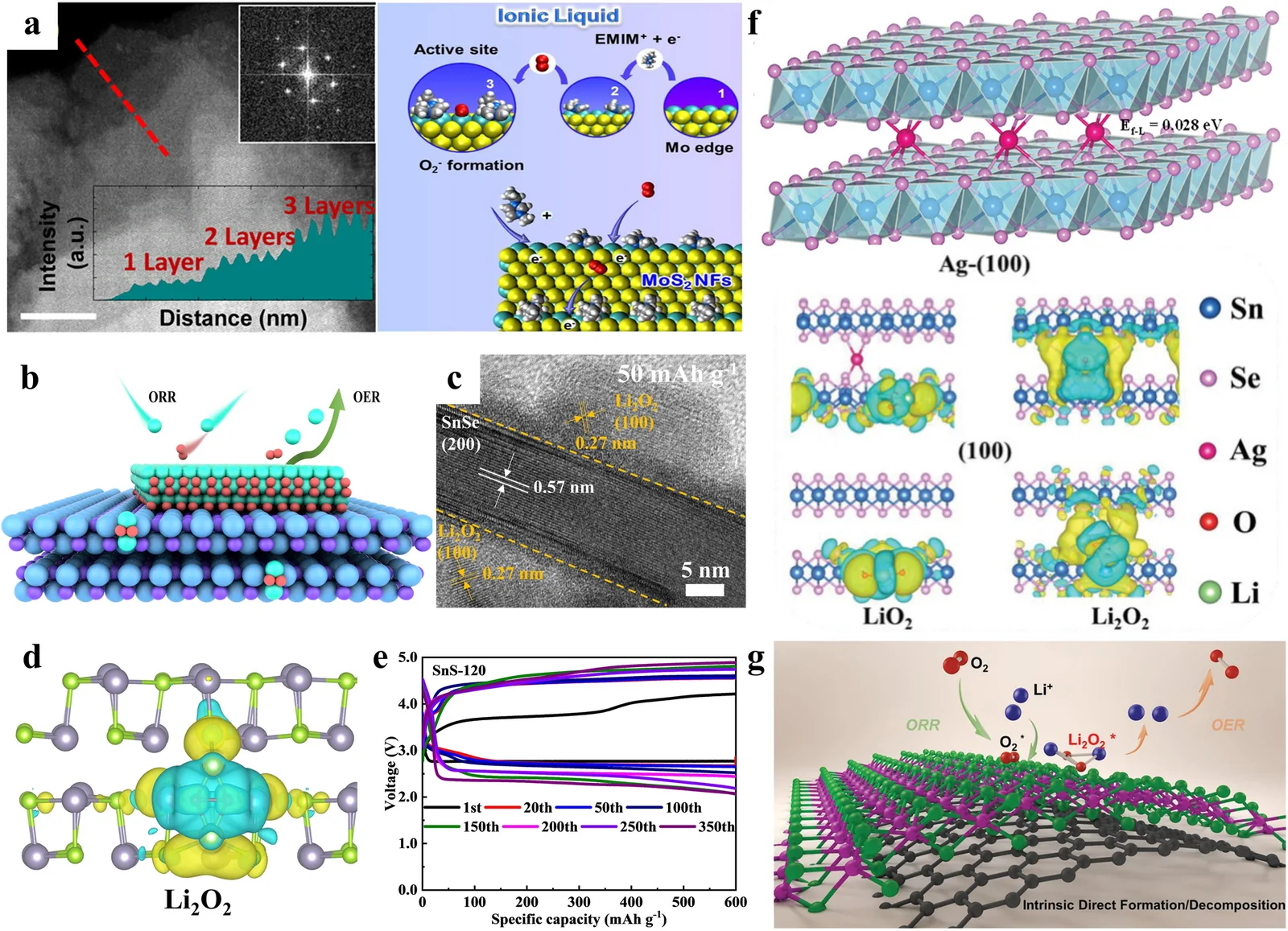
**a** STEM image of MoS_2_ nanoflake and edge protection strategy [183]. **b** Schematic illustration of catalytic mechanism on the SnSe surface. **c** TEM image of initial discharge products on the SnSe surface. **d** Charge density difference of Li_2_O_2_ adsorbate on the SnSe edge plane. **e** Electrochemical performance of SnSe cathode with limited capacity of 600 mAh g^−1^ and current density of 500 mAh g.^−1^ [187]. **f** Illustration of Ag-doped SnSe_2_ and charge density difference after adsorbed reaction species [185]. **g** Schematic illustration of the direct formation/decomposition of Li_2_O_2_ on the surface of MoSe_2_ [186]

TMDs, bearing resemblance to graphene, possess unique electron transfer properties for edge and basal planes, in which the conductivity of the edge direction greatly surpasses that of basal plane. Indeed, this phenomenon induces anisotropy on the two planes in catalytic processes [11, 12]. It is well known that the edge metal Mo atoms with high electron conductivity serve as catalytic sites for MoS_2_ during HER process [181]. However, this situation appears to be totally distinct in the cathode reaction of LOBs involving the ORR and OER processes. The edge metal Mo atoms in MoS_2_ exhibit a strong adsorption affinity toward oxygenated species accompanying the dissociation of O–O bond. This process not only deactivates the edge sites by oxidation but also obstructs the decomposition of the product due to high adsorption strength [182, 183]. Asadi et al. applied the EMIM^+^ ionic-liquid electrolyte to the LOBs, in which the EMIM^+^ molecules randomly cover the edge Mo sites, thus preventing the direct dissociation of O_2_ by two adjacent exposed Mo atoms (Fig. 9a) [183]. In their subsequent work, this approach enabled the MoS_2_ catalysts to exhibit excellent electrochemical performance in a simulated air atmosphere with a long cycle life of up to 700 cycles [184].

In following, Dang group conducted a series of work to thoroughly study the correlations in stacked layer structure and catalytic anisotropy of 2D selenides [185–188]. Specifically, for SnSe with simple two-atom layer structure in one stacking unit, the 2D SnSe features facet-dependent selective Li_2_O_2_ growth in the discharging process (Fig. 9b, e) [187]. The results demonstrate that the growth of products is initially concentrated on the surface plane, rather than the stacking edge plane, because the absence of electrons in layer space on edge plane disturbs the structure of Li_2_O_2_ and restricts the nucleation and growth of products. In addition, the SnSe_2_ with three-atom layer further presented selective catalytic properties [185]. Notably, the Ag ions are intercalated into the SnSe_2_ layer space, which can compensate the rare electron in layer space and act as electronic bridge in increasing the interaction and diminishing catalytic anisotropy (Fig. 9f). The layer number is another factor for catalytic capability of 2D TMDs. The few layer MoSe_2_ displays intrinsic catalytic properties and promote the direct formation/decomposition of Li_2_O_2_ [186]. Previous research has shown that the step edge structure of anisotropic Pt catalysts has shown enhanced ORR/OER performance in LOBs [189]. However, for TMDs catalysts in LOBs, more studies are needed to elucidate the catalytic activity about the step structure with coexistence of edge and basal plane.

#### Electronic Modulation and Structural Designment

**Fig. 10:**
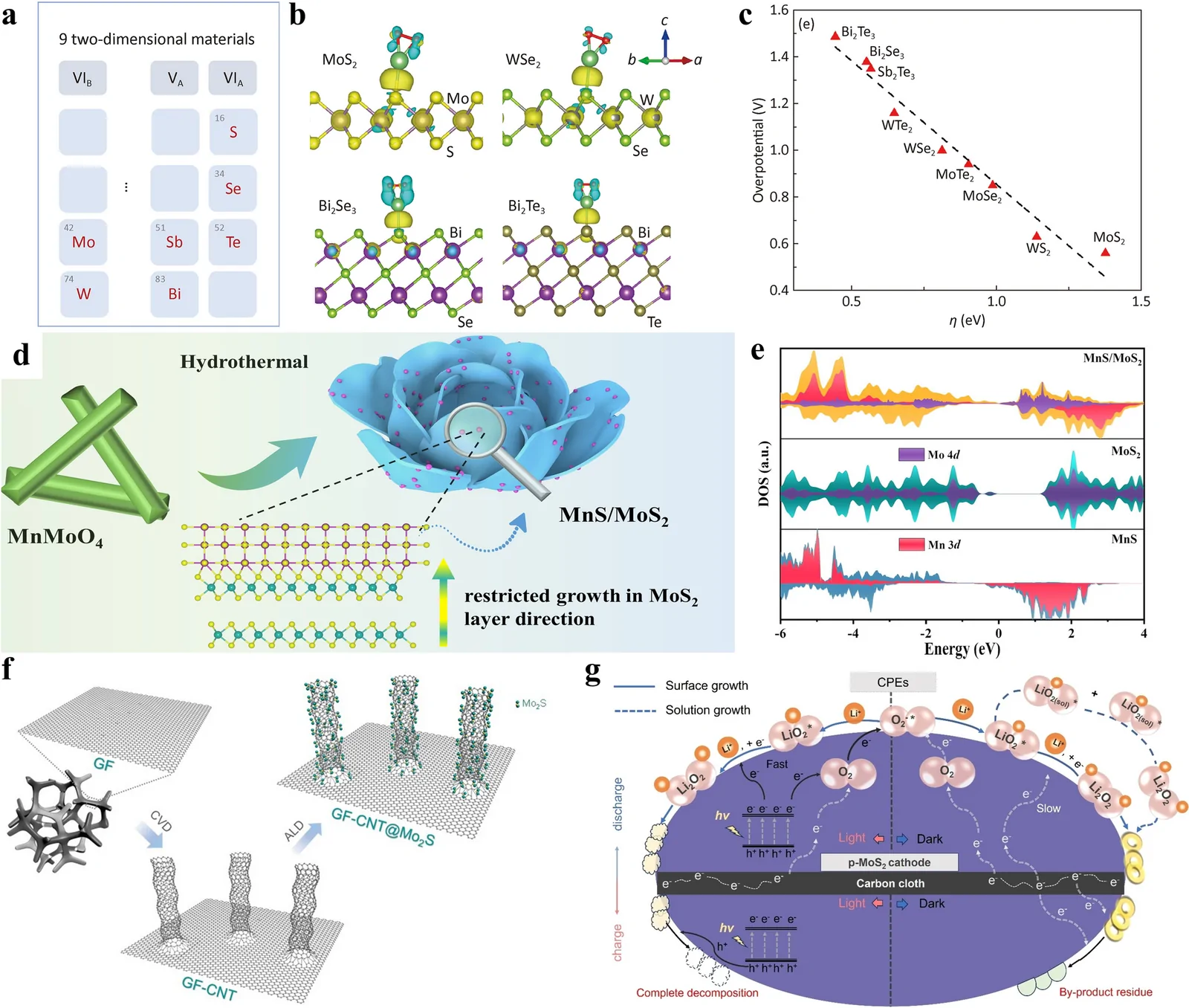
**a** Nine 2D materials composed of Group VIB, VA, and VIA elements.** b** Differential charge density of LiO_2_ adsorbed on typical 2D materials. **c** Trend chart of overpotential variation with catalytic merit value η for 9 materials [191]. **d** Schematic illustration of the fabrication procedures of MnS/MoS_2_ heterostructure. **e** Density of states of different catalysts [28]. **f** Fabrication of GF-CNTs@MoS_2_ [198]. **g** Illustration of the light assistance effect for the growth mechanisms of Li_2_O_2_ on MoS_2_ surface [200]

2D TMDs have been widely studied as cathode catalysts in LOBs; nevertheless, most of the highly efficient TMDs require further adjustment and modification to improve the thermodynamic and kinetics processes [179, 190, 191]. Recently, Cheng et al. calculated the theoretical catalytic performance of nine typical 2D materials for LOBs (Fig. 10a–c). The results indicate that MoS_2_ with appropriate Li–X (X represents chalcogen elements) bond energy and lattice constant has the best electrochemical performance [191]. In addition, constructing the non-metal vacancies and defects in TMDs can adjust the adjacent atomic arrangement and electronic structure. For instance, the S vacancy in MoS_2_ exposes more active inner Mo sites and increases the electron transfer, thereby affecting the discharge products and exhibiting superior electrocatalytic activities [62, 126]. This process becomes more pronounced with the increase in the concentration of S vacancies [192]. In TMDs, the high similarity of non-metal S and Se atoms enables them to be easily and controllably incorporated into the same material for the regulation of electronic structure [193–195]. Zhang et al. prepared metastable MoSSe solid solution to distort the lattice structure and tune its catalytic activity for the LOBs [193]. It is found that properly tailored atomic structure in MoSSe is to be efficient on significantly improving the electrochemical activity and facilitating the fast ion transport.

The electronic modulation can also be well realized on a large scale by constructing heterostructures in 2D TMDs. Recently, some MoS_2_-based heterostructures have been reported as cathode catalysts in LOBs, such as those incorporated with NiS_2_ [29], and MnS [28]. Due to the synergistic effect among different components, the TMDs heterostructures present improved electrochemical performance via E_g_ orbital modulation, d-band center regulation as well as the adsorption strength optimization. Specifically, the incorporation of MnS within the MnS/MoS_2_ heterostructure avoids the agglomeration of MnS and restricts the stacking growth of MoS_2_ (Fig. 10d) [28]. Consequently, with the assistance of the built-in electrical field, the well-defined MnS/MoS_2_ heterostructure increases electron densities and expedites the electron transfer (Fig. 10e), which optimizes the adsorption strength of reaction species and finally modulates the reaction pathways. Long et al. showed that the heterogeneous interface in Ni_0.85_Se/MoSe_2_ is highly matched to the Li_2_O_2_ (100) plane, facilitating epitaxial growth of Li_2_O_2_, as well as the decomposition of products during the cycles [127]. In addition, phase modulation engineering is another method by building heterojunctions to boost catalytic activity. The combination of 1 T/2H MoS_2_ utilizes the high electronic conductivity of metallic 1 T phase and good stability of 2H phase, synergically improving the electrochemical performance of LOBs [37, 196].

Additionally, considering the triple-phase interface in the cathode reaction, the catalyst morphology plays a key role in Li^+^ transport and O_2_ diffusion, determining the rate performance and discharge capacity of LOBs. Currently, the morphologies of nanoflowers [28, 197] and nano-flakes/sheets [182, 193] for MoS_2_ have been widely studied in the LOBs. Furthermore, conductive additives, such as graphene, CNTs, and carbon spheres, are also applied into the synthesis process to diversify the morphology designs [62, 126, 186]. The conductive additives contribute to a large surface area and high electron conductivity, and these provide more sites and spaces for discharge product deposition. In turn, the active TMDs coverage prevents the carbon materials from electrolyte corrosion. Song et al. fabricated an integrated cathode that contains an amorphous MoS_2_ thin layer deposited on 3D conductive carbon scaffold (Fig. 10f) [198]. The highly conductive, lightweight, and macropore GF-CNTs@MoS_2_ network has facilitated ions/gas transport, maintained the high activity during long cycles, and offered mechanical flexibility of the LOB cell. Given that TMDs are typical semiconductive materials, the implementation of external field-assisted strategies will efficiently improve the reaction kinetics in LOBs [38]. For instance, Tian et al. utilize the piezoelectric effect of MoS_2_/Pd nanocomposites and establish force-field-assisted LOBs systems by concerting the mechanical energy from ultrasound [39]. Wei et al. fabricated freestanding WSe_2_ nanofiber and facilitated the charge carrier under light assistance, thereby enhancing the electrochemical performance in LOBs [199]. Furthermore, Ren et al. proved that light induced a change of reaction pathways on the MoS_2_ surface, which benefited from the fast response triggered by photogenerated electrons and holes during working processes [200]. In consequence, the spherical-like Li_2-x_O_2_ particles were generated via single surface growth path under illumination and were fully decomposed without formation of by-products in the following charging process (Fig. 10g).

### Other 2D Structure Materials

Considering special advantages of 2D structures, some other advanced 2D materials have been reported in LOBs from theoretical predication and experimental tests. The electrocatalytic capability of single atomic layer materials, such as silicene [201], phosphorene [202], and germanium [203] was evaluated by theoretical calculations. Among them, silicene was prepared from Si powder through continuous lithiation/delithiation methods and tested as cathode catalyst in LOBs [204]. Furthermore, MOFs, which are comprised of organic ligands coordinated to various metal ions, are a type of crystalline porous polymer networks. Ultrathin MOF sheets display a spatially infinite network configuration in two-dimensional directions and have attracted much attention as catalysts because of their merits of large specific surface area, high porosity, and rich exposed redox-active centers [205]. Meanwhile, the 2D structure favors shortening ion diffusion distance and increasing the carrier concentration and conductivity, which further enhance the electrochemical performance. The first wide application of MOFs is their derived carbon-based materials, which possess tunable morphology and accessible metal sites and enable them to become great candidates for the cathode catalysts in LOBs [114, 119, 122, 123]. Here, besides MOFs derivatives, we mainly concentrate on the pristine 2D MOFs materials and Lin et al. reviewed their intrinsic catalytic properties [205].

In the context of 2D MOFs, the metal sites normally serve as the catalytic centers and exert influence on the catalytic performance by regulating the electronic structure, moderating the adsorption strength, and changing the reaction paths [206–211]. For instance, Lv and colleagues demonstrated that increasing the spin state of Ni metal in Ni-MOF can facilitate electron exchange between the Ni sites and oxygen adsorbates and accelerate the oxygen redox kinetics [207]. MOFs with three different metal clusters (Fe, Ti, and Zr) were synthesized by Tao et al. and applied as cathode catalysts in LOBs [208]. The results indicate that Fe-MOF exhibits a large surface area and excellent O_2_ adsorption ability, which contributes to long cycle life of 195 cycles and high energy efficiency of 93%. In addition, the rich pores in 2D MOFs provide mass transfer pathways for reaction and influence the reaction kinetics. Min et al. proposed an encapsulation mechanism, in which the pore size of 2D MOFs shows a positive correlation with the trapping number of ions, thus influencing the adsorption energy of ions as well as the conductivity of the system [212]. Wang et al. extended the pore size from 1.8 to 3.2 nm by inserting another ligand to build linear M-ligand-M units [213]. The electrochemical analysis demonstrates that the conjugated MOF with large pore size facilitates the diffusion of redox species. However, although remarkable progress has been made in the study of 2D MOFs for electrocatalysts, the low conductivity due to organic ligands restricts further development. The low stability of 2D MOFs to electron beam also brings challenges to the observation of catalytic sites. Therefore, the future potential solutions should lie in rationally designing the structure and establishing structure–activity relationships to fully exert their catalytic capability.

## Mechanistic Perspective across 2D Cathode Catalysts for Li–O_2_ Batteries

Despite the diverse materials categories of 2D catalysts, a comprehensive understanding based on fundamental elemental properties and basic reaction mechanisms in LOBs will accelerate the establishment of a unified mechanistic framework that links intrinsic electronic structure, intermediate adsorption, and catalytic performance. The intrinsic electronic structure of 2D materials governs the adsorption strength to key intermediate (e.g., LiO_2_), which determines the thermodynamics of intermediate stabilization and decomposition. Meanwhile, the solvent effect should be explicitly considered, as they balance the stabilization and dissociation of LiO_2_ intermediates in the electrolyte. Therefore, in this section, we summarize the electrochemical performance of 2D catalysts in LOBs, with an emphasis on the important role of machine learning in guiding experimental design and establishing structure–property relationships. In addition, advanced in situ characterization techniques are highlighted to provide more direct insights into the reaction mechanisms. Furthermore, theoretical model construction is also introduced to enable more comprehensive information.

### Catalytic Principles and Activity Descriptors of 2D Catalysts

**Fig. 11:**
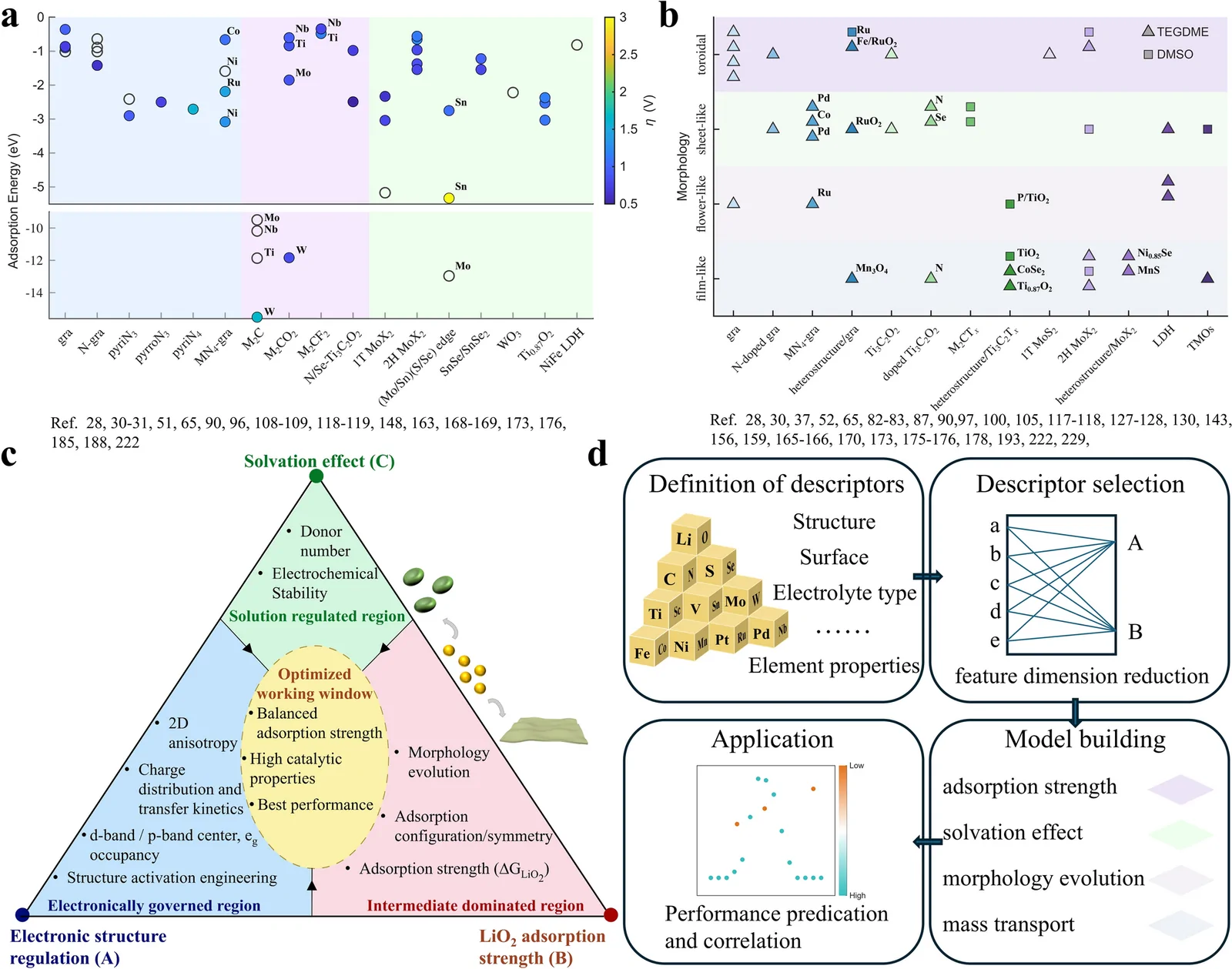
**a** Summary and classified LiO_2_ adsorption energies on different substrates, where the heat-map color represents the calculated overpotential. **b** Summary and classified Li_2_O_2_ morphologies on different substrates. **c** A ternary phase diagram-inspired mechanistic framework in LOBs. **d** A general workflow for the application of machine learning in LOBs

Bruce et al*.* established two Li_2_O_2_ formation pathways—surface growth and solution-mediated growth—which has been widely adopted as a fundamental framework in subsequent studies [54]. According to the theory, the adsorption energy of LiO_2_ on the electrode surface is often selected as descriptor to explore the disparate morphology and structure of Li_2_O_2_ [214, 215]. To better compare the adsorption behavior of different 2D catalysts, we selected and classified representative 2D materials and plotted the relationship between adsorption strength of LiO_2_ and overpotential. As shown in Fig. 11a, N-doped graphene strengthens the LiO_2_ adsorption by introducing extra electrons and modulating coordination [105, 106]. In LOBs, a proper adsorption strength is necessary for reversible formation/decomposition products. It should be noted that metal atom-doped graphene demonstrates a much broader modulation range of adsorption strength compared to heteroatom-doped systems. This behavior arises from the participation of d orbitals, which endow the active sites with multiple tunable features, including electronic structure, coordination geometry, and valence states. As a result, metal-doped systems provide an expanded adsorption descriptor space, enabling access to different catalytic regimes. For MXene materials, surface terminal groups play a key role in determining their physicochemical properties. The terminal groups in MXenes are an integral part of the surface structure and directly participate in adsorption and catalysis. They strongly modulate the electronic structure, charge distribution, and coordination environment of transition metal sites, thereby significantly affecting the adsorption strength of reaction intermediates. When the target shifts to morphology of discharge products (Fig. 11b), the situation becomes different and varied morphologies are observed on the cathode. It reveals the gap between the calculation and experiential results, which can be attributed from the solvation effects and other experimental optimization parameters. The summary of the typical 2D catalysts used in LOBs is provided in Table 1. Khetan et al. demonstrated that solvent stability, proton/hydrogen abstraction via nucleophilic attack, and the ability to facilitate solution-mediated discharge collectively determine battery performance, with appropriate solvent environments leading to enhanced discharge capacities [216].

**Table 1:** Selective summaries of 2D cathode catalysts used in LOBs

2D catalysts	Current (mA g^–1^ or mA cm^–2)^	Capacity (mAh g^–1^ or mAh cm^–2^)	Cycle number	Descriptor	Modification strategies	Electrolyte	Product morphology	Refs
Graphene quantum dots	1400	68,900	300	structural/edge defect density	edge engineering (2)	LiTFSI/TEGDME	toroidal	[79]
Graphene quantum dots	0.1	7672	500	electronic conductivity	edge engineering (2)	LiTFSI/TEGDME	flower-like	[80]
GMS	0.2	6727	70	LiO_2_ adsorption energy	topological defect (2)	LiTFSI/TEGDME	toroidal	[95]
Porous graphene	200	7400	400	mass transfer efficiency	porous architecture (3)	LiTFSI/TEGDME	toroidal	[93]
100N GMS	0.4	23	185	number of stacking layers	stacked graphene (3)	LiTFSI/LiNO_3_ TEMPO/TEGDME	toroidal	[97]
PdN_4_-graphene	200	10,000	60	d-band center and LiO_2_ adsorption energy	single-atom Pd doping (1)	LiTFSI/TEGDME	sheet-like	[114]
RuN_4_-graphene	0.02	12,724	–	LiO_2_ adsorption energy	single-atom Ru doping (1)	LiTFSI/TEGDME	flower-like	[115]
Ru@graphene	200	21,753	200	mass transfer	ruthenium-modified (2)	LiNO_3_ in DMSO	toroidal	[245]
Fe_SA_-RuO_2_/HPCS	200	23,628	232	d-band center and LiO_2_ adsorption energy	Fe doping (1)	LiSO_3_CF_3_/TEGDME	toroidal and sheet-like	[124]
Mn_3_O_4_@graphene	200	35,583	130	Li_2_O_2_ adsorption energy	surface engineering (2)	LiTFSI/TEGDME	film-like	[125]
CoN_4_-graphene	100	13,544	98	LiO_2_ adsorption energy	single-atom Co doping (1)	LiTFSI/TEGDME	Sheet-like	[246]
Ti_3_C_2_ QDC/N–C	200	16,022	240	d-band center	quantum dot and heterostructure (2)	LiTFSI/TEGDME	flower-like	[160]
Ti_2_CO_2_ MXene	100	15,635	250	Li_2_O_2_ adsorption energy	surface modification (2)	LiNO_3_/DMSO	sheet-like	[161]
N-Ti_3_C_2_O_2_	100	11,679	372	3d orbit occupancy	N doping (1)	LiTFSI/TEGDME	film-like	[167]
Ti_0.87_O_2_/Ti_3_C_2_	100	13,596	407	LiO_2_ adsorption energy	heterostructure (3)	LiTFSI/TEGDME	film-like	[173]
P-doped TiO_2_/Ti_3_C_2_T_x_	100	13,482	190	Li_2_O_2_ adsorption energy	P-doping (1)	LiNO_3_/DMSO	flower-like	[174]
Ti_3_C_2_/Mo_4/3_B_2-x_ superlattice	100	17,167	475	d-band center	superlattice (2)	LiTFSI/TEGDME	film-like	[175]
F-Nb_2_C MXene	200	19,785	130	LiO_2_ adsorption energy	O-terminated (2)	LiNO_3_/DMSO	sheet-like	[30]
6Ag-SnSe_2_	100	16,871	144	LiO_2_ and Li_2_O_2_ adsorption energy	Ag Intercalation (1)	LiNO_3_/DMSO	sheet-like	[185]
MoS_2-x_	200	19,989	355	LiO_2_ and Li_2_O_2_ adsorption energy	sulfur defect (1)	LiTFSI/TEGDME	film-like	[62]
MnS/MoS_2_	100	11,696	248	LiO_2_ and Li_2_O_2_ adsorption energy	heterostructure (3)	LiTFSI/TEGDME	film-like	[28]
1 T MoS_2_	100	7441	622	Electronic structure	phase transition (3)	LiTFSI/TEGDME	toroidal	[37]
MoSe_2_	100	32,576	270	LiO_2_ and Li_2_O_2_ adsorption energy	core–shell structure (3)	LiNO_3_/DMSO	sheet-like	[186]
Ni_0.85_Se/MoSe_2_	100	15,994	175	Li_2_O_2_ adsorption energy	heterostructure (2)	LiTFSI/TEGDME	film-like	[127]
Ni/Mn-MoS_2_	100	28,195	118	LiO_2_ adsorption energy	Ni-Mn co-doping (1)	LiNO_3_/DMSO	film-like	[197]
Def-MoS_2_	0.1	6.7	100	O_2_ adsorption energy	sulfur defect (1)	LiClO_4_/DMSO	film-like	[192]
NiCo-LDHs-V_O_	200	26,853	100	Electronic structure	oxygen vacancy (1)	LiTFSI/TEGDME	flower-like	[152]
CoNi-LDH	200	10,664	209	Layer structure	terephthalic acid intercalation (2)	LiTFSI/TEGDME	flower-like	[155]

The numbers (1–3) in the “Modification strategies” column denote different activation engineering approaches: (1) point activation engineering; (2) line and plane activation engineering; and (3) bulk activation engineering

In Fig. 11c, a ternary phase diagram-inspired mechanistic framework was adopted to connect electronic structure, intermediate adsorption, solvation effect, and electrochemical performance. Various activation strategies work in electronic structure regulation region, where they modulate electronic behavior and develop various orbital descriptors to explain the subsequent catalytic performance. As a result, this region governs the subsequent reaction evolution, particularly the behavior to key reaction intermediates (intermediate dominated region), such as LiO_2_, whose adsorption strength and configuration dominate the reaction pathways and products morphology evolution, thereby determining the capacity performance. Meanwhile, the solvation effect modulates the stability of LiO_2_ in the electrolyte, thereby influencing Li_2_O_2_ morphology and mass transport, while electrolyte stability is critical for suppressing side reactions and maintaining system reversibility. Overall, through the rational regulation of these regions, an optimal working condition can be achieved, leading to best electrochemical performance in LOBs. However, given the multi-dimensional parameter space and massive data sets, it is a great challenge to establish quantitative structure-electronic-intermediate–performance relationships. Machine learning, a very powerful data-driven approach and subset of artificial intelligence, is accelerating this analytic process. Recently, Liu et al. employed classical machine learning algorithms combined with Bayesian optimization to explore dual-solvent (DMSO/TEGDME) and single-salt (LiTFSI) electrolyte systems [217]. The data-driven results revealed that an optimal electrolyte composition of DMSO/TEGDME 97.6%/2.4% with a LiTFSI concentration of 0.47 M can effectively reduce the Li^+^ desolvation energy and enhance battery performance. Zhang et al. integrated high-throughput workflow and machine learning framework to systematically investigate MXene-based catalysts in LOBs and identified the exceptional catalysts Mo_3_C_2_Cl_2_ with low overpotential 0.01 V [218]. Aysegul et al. collected 1015 data points from the literature, which were then analyzed using association rule mining and decision tree algorithms [219]. A comprehensive set of factors was considered, including the anode, separator, reactant, operating pressure, gas diffusion layer, catalyst, binder, mass loading, and electrolyte. Association rule mining and decision tree algorithms show that bulk cathode materials, especially N-doped carbons, graphene, and porous carbons, are beneficial for achieving high performances. Figure 11d displays a general workflow for the application of machine learning in LOBs, which includes data collection, descriptor selection, model building, and prediction. Several key descriptors, including facet structure, charge distribution, adsorption energy, and electrolyte properties, play a critical role in predicting catalytic performance. Nevertheless, further studies result about machine learning in LOBs are still required to establish a clear and unified mechanistic understanding.

### Advanced Analytic Techniques

**Fig. 12:**
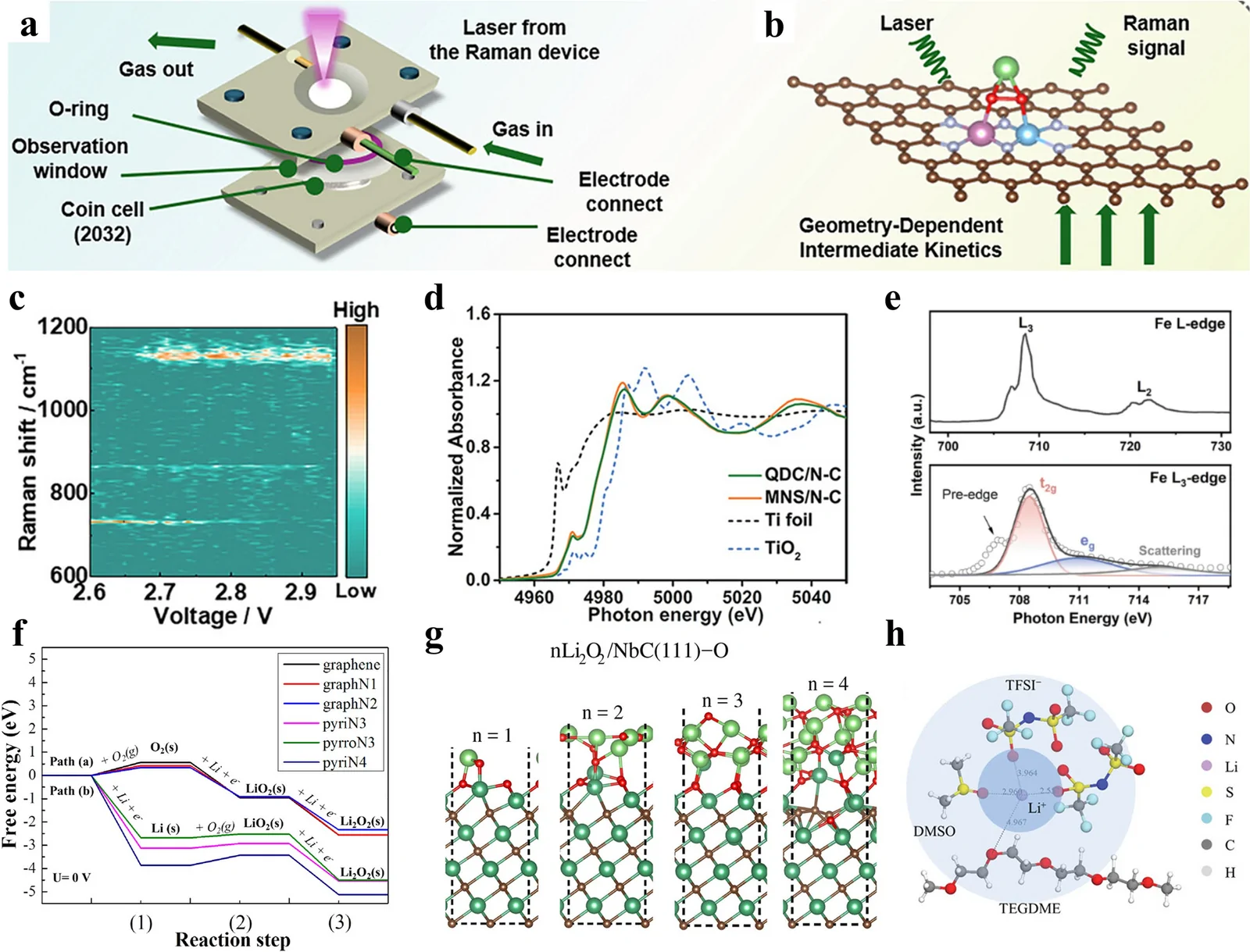
**a** Schematic of in situ Raman spectroscopic model. **b** Schematic diagram of the in situ Raman spectroscopic monitoring. **c** Time-resolved in situ Raman 3D image of TiIn–N–C catalysts [221]. Copyright 2026, John Wiley and Sons. **d** Normalized K‐edge XANES of Ti_3_C_2_ QDC/N–C, Ti_3_C_2_ MNS/N–C, and Ti–foil, TiO_2_ references [160]. Copyright 2021, John Wiley and Sons. **e** Fe L-edge and L_3_-edge XANES spectra for FeTiO [227]. Copyright 2026, Elsevier. **f** Calculated energetic profiles of the initial nucleation of Li_2_O_2_ on different surfaces [105]. Copyright 2015, American Chemical Society. **g** Lowest energy structure of nLi_2_O_2_ on the NbC surface [228]. Copyright 2020, American Chemical Society. **h** Schematic of the interaction between solvent molecules and Li ions [229]. Copyright 2021, John Wiley and Sons

A rational design of catalysts combined with the leverage of advanced analytical techniques can significantly deepen our understanding of reaction mechanisms. Here, several recent advanced characterization techniques are introduced for probing the reaction processes and electronic structures. Raman spectroscopy, as an ultrasensitive characterization technique with single-molecule-level sensitivity, plays a powerful role in understanding interfacial electrochemistry. Based on the inelastic scattering of photons, it can sensitively detect the formation of intermediates and products such as LiO_2_ and Li_2_O_2_ by probing molecular vibration [220]. Figure 12a shows a representation in situ Raman cell model; incident laser penetrates the optical window reaching the surface of work electrode. Guan et al. used in situ Raman spectroscopy to elucidate the catalytic roles of geometry and spacing of dual-atom catalytic centers (Fig. 12b, c) [221]. The broad peak centered at approximately 1129.8 cm^−1^ was identified as the LiO_2_ intermediate. These results show that the TiIn–N–C exhibited an earlier LiO_2_ emergence window and more efficient Li_2_O_2_ formation than single In–N–C catalyst, which demonstrate that the introduction of Ti sites reshapes the orbital interactions and coordination environment, thereby enabling improved electrochemical performance. Furthermore, Zhang et al. monitor the reversible nucleation and decomposition of Li_2_O_2_ (808 cm^−1^) throughout the discharge/charge cycle. The E_g_ and F_2g_ orbitals of Co metal/oxide exhibit splitting firstly, indicating strong O_2_ adsorption in an oxidizing environment [220]. Despite the clear spectral distinctions, Raman spectroscopy still faces challenges in signal sensitivity as well as spatial and temporal resolution. Meanwhile, the spectral results are primarily qualitative and often lack quantitative information regarding the reaction processes.

Differential electrochemical mass spectrometry (DEMS) is another powerful analytical technique that enables quantitative assessment of gas species and compositions during reaction processes [222]. In the field of LOBs, DEMS serves as a pivotal tool for elucidating reaction mechanisms, identifying parasitic reaction pathways, and evaluating electrolyte stability. The charge-to-mass ratio (e^−^/O_2_) obtained from the gas evolution platform can be categorized into three distinct scenarios: ratios approaching 1.0, close to 2.0, and exceeding 2.0, which correspond to the formation of soluble LiO_2_, the decomposition of insoluble Li_2_O_2_, and contributions from additional side reaction, respectively [223]. Such differentiation allows for effective determination of reaction pathways and the occurrence of irreversible side reactions; however, these side reactions can also be directly monitored by detecting the release of gases such as CO_2_ and H_2_. When combined with fluorescent probes, such as dimethylacetamide for singlet oxygen detection or isotopic labeling techniques—such as using ^13^C-labeled electrodes and 18O_2_ atmospheres, it reveals the pathways of byproduct gas release, indicating degradation of carbon cathode and organic electrolytes under electrophilic attack by highly reactive oxygen species [224, 225].

Synchrotron K-edge X-ray absorption spectroscopy (XAS) technique can probe the local electronic and geometric structures of specific elements by measuring the absorption coefficient as a function of X-ray energy. The absorption spectrum is typically divided into two regions: X-ray absorption near-edge structure (XANES) and extended X-ray absorption fine structure (EXAFS). XANES is highly sensitive to the electronic states of the atom and is widely employed for valence state analysis. The absorption edge position exhibits a characteristic shift depending on the oxidation state, enabling the valence of the target element in an unknown sample to be determined through comparison with reference compounds. Wang et al. demonstrated that the white-line peak energy of Ti_3_C_2_ QDC/N–C is positively shifted in reference to Ti_3_C_2_ MNS/N–C due to the more positively charged state of Ti atoms (Fig. 12d) [160]. Importantly, XANES signals are strong and exhibit excellent penetration, making this technique particularly suitable for analyzing samples with low dopant concentrations or defects, as well as for real-time monitoring of valence state changes in two-dimensional materials during electrochemical cycling in batteries. EXAFS offers atomic-scale resolution of the coordination environment by analyzing the oscillatory pattern in the energy range of 50–1000 eV on the high-energy side of the absorption edge. Zhang et al. demonstrated successful doping of single-atom platinum sites in the Mo_2_TiC_2_T_x_-PtSA system, as no Pt–Pt bonds were detected in the EXAFS fitting results and only three Pt–C bonds were present [169]. The synchrotron L-edge X-ray absorption spectroscopy arises from electric dipole-allowed transitions of 2*p* electrons to unoccupied d orbitals (2*p*^6^3*d*^n^ → 2*p*^5^3*d*^n+1^). The resulting spectra exhibit two characteristic absorption edges, L_3_ and L_2_, due to spin–orbit coupling of 2*p*^5^ core configuration. The sharp “white-line” feature makes L-edge spectroscopy a powerful tool for directly probing the d-electronic states of transition metals and lanthanides. Although this technique requires high vacuum conditions and soft X-rays have limited penetration depth, the intensity of L-edge transitions is directly related to the *d*-character of the metal in unoccupied valence orbitals. Consequently, L-edge spectroscopy is highly sensitive to the oxidation states, spin states, and crystal field splitting of elements, providing a more precise elucidation of local electronic structures and reflection of metal–ligand covalency compared to K-edge techniques. Yao et al. identified the occupied d orbital states (t_2g_ and e_g_) of Ni in a typical octahedral field and the further splitting of Mn 3*d* orbitals (*d*_xz/yz_, *d*_xy_, *d*_z_^2^, *d*_x_^2^-_y_^2^) in a distorted octahedral field via L_3_ edges for Ni and Mn in Ni–MnO_2_. The observed blueshift in the Ni L_3_-edge indicates an increase in Ni’s oxidation state, while the redshift in the Mn L_3_-edge reflects a decrease in Mn oxidation state [226]. In addition to oxidation states, Zhang et al. determined the intermediate spin state of Fe in Ti_0.6_Fe_0.4_O_2_ by calculating the area ratio of the split peaks (e_g_ and t_2g_) in the Fe L_3_-edge spectrum (Fig. 12e) [227].

### Density Functional Theory Model Construction

Theoretical calculations by DFT-based first-principles calculation enable in-depth understand of the reaction of mechanists at atomic level from inherent natures of materials. 2D materials exhibit a characteristic layered structure, which facilitates the rapid identification of the exposed crystal planes—the basal plane (in-plane direction) and the edge plane (interlayer termination direction). The differences in adsorption behaviors exhibited by the basal and side planes are crucial for exploring the catalytic anisotropy of 2D materials. It presents that the catalytic anisotropy of 2D SnSe originates from the uniform adsorption of Li_2_O_2_ on the basal plane (200) and the confined adsorption on the side plane (002), as revealed through a comparative analysis of adsorption sites and energies [187]. As shown in Fig. 12f, adsorption energy as an important result provides insights into the feasibility of adsorption processes, determining the design of later reaction pathways. Compared with Li_2_O_2_ molecules, small (Li_2_O_2_)_n_ cluster will be better to describe the early state of nucleation and growth of discharge products. Figure 12g presents the structural relaxation of MXene materials with increasing Li_2_O_2_ layers. The Li–O bonds in the first Li_2_O_2_ layer are broken due to the strong interaction with the surface, leading to the formation of Li/O interfacial layers rather than intact Li_2_O_2_ molecules [228]. As additional Li_2_O_2_ layers accumulate, the initially formed interface becomes buried, and the system gradually evolves toward bulk-like Li_2_O_2_ growth, where structural distortions are reduced and the film ultimately approaches an insulating state. In addition, considering the significant influence of solvent effects on reaction pathways, solvation should be taken into account (Fig. 12h) [229]. However, the explicit inclusion of solvent molecules greatly increases computational cost. Therefore, simplified approaches, such as implicit solvation models or molecular dynamics simulations, are often employed to balance accuracy and computational efficiency.

## Functional Extension of 2D Materials for Li–O_2_ Batteries System

**Fig. 13:**
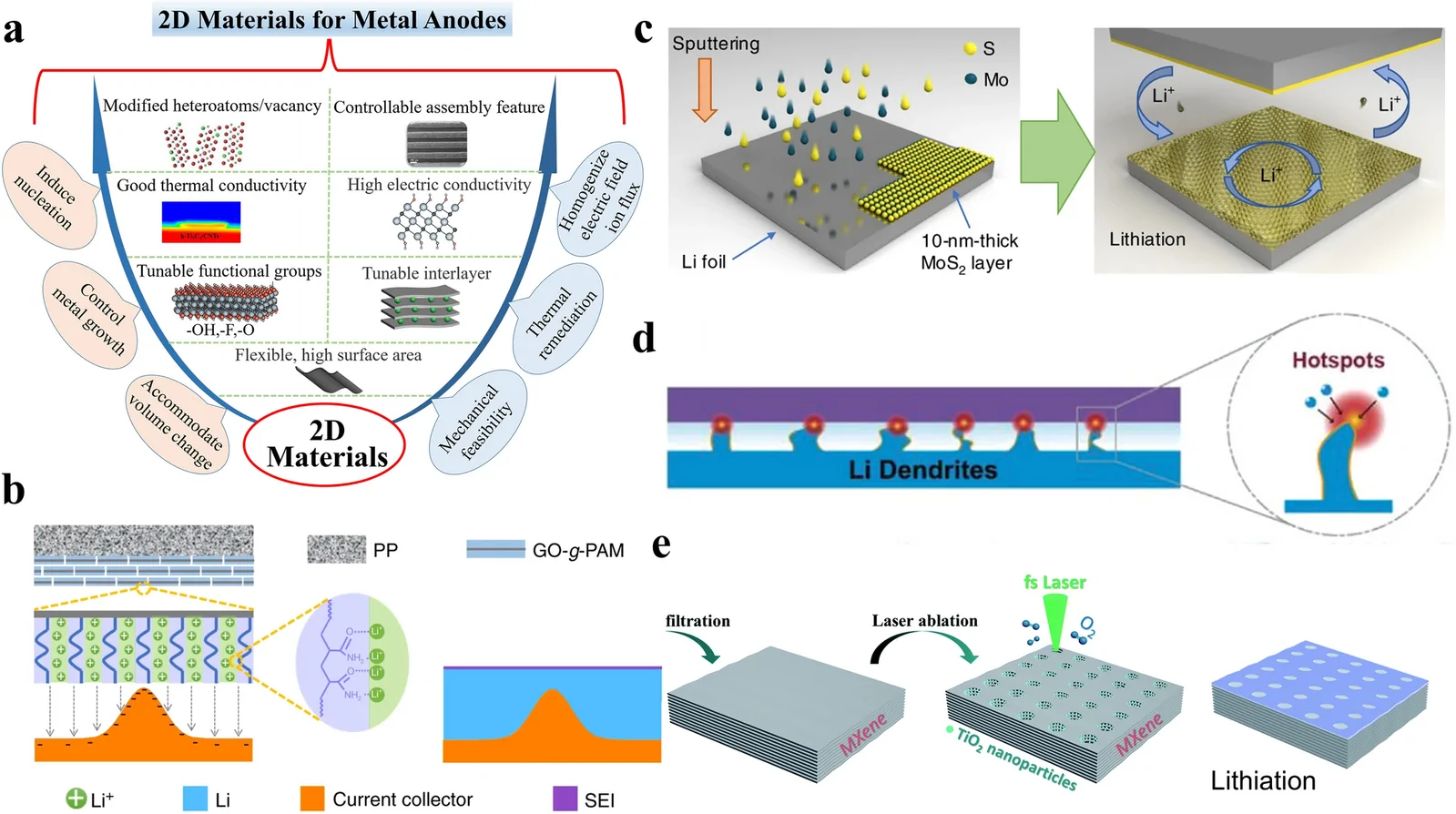
** a** Overall diagram of harnessing the unique properties of 2D materials toward metal anodes [43]. Copyright 2021, John Wiley and Sons.** b** Schematic illustration of graphene oxide backbones suppressing the Li dendrite [236]. Copyright 2019, Springer Nature.** c** Schematics illustrate the fabrication method for a MoS_2_-coated Li anode via sputtering and subsequent lithiation [237]. Copyright 2018, Springer Nature.** d** Study of lithium deposition behavior under localized-temperature hotspots [239]. Copyright 2022, John Wiley and Sons.** e** Schematic illustration of the fabrication process of Ti3C2Tx MXene and lithiation morphology [241]. Copyright 2020, Royal Society of Chemistry.

With the in-depth study and increasing application requirements, it has been acknowledged that not only the active 2D catalysts, but also the functionality and coordination of various components, including electrolyte, separator, and Li anode, are crucial for enhancing the overall performance of the LOBs [43, 44, 49]. Particularly, the first concern is the formation of Li dendrites caused by irreversible stripping and deposition of lithium ions during cycling processes, which leads to the decrease in capacity, cycling life, and short circuit. Moreover, the physicochemical properties of electrolyte and separator are also necessary for the further improvement of the system. Fortunately, certain 2D materials play a key role in anode and separator protection owing to their outstanding mechanical properties, such as high Young’s modulus and structural flexibility, which enable them to withstand high stress and strain without structural degradation. In addition, the uniform surface structure and tunable surface chemistry of 2D materials facilitate ion transport by reducing the dissociation energy of Li^+^ and weakening cation–anion interactions in the electrolyte, thereby enhancing ionic conductivity (Fig. 13a) [43, 73] Therefore, in this section, we mainly introduce the extended function of 2D materials in the LOBs system beyond cathode catalysis.

### Mechanical Strength and Thermal Stability

Lithium dendrite is one of main challenges for lithium metal during the long-time cycling process. It is reported that the high elastic modules of lithium dendrite (4.0 GPa) can easily puncture the common separator (e.g., 2.1 GPa for polyolefin-type separators), leading to shortcut and safety issues in batteries [43, 49]. Introducing the protective layer in the anode side with high mechanical strength and fast ion transport capability is direct and effective approaches to address these problems. It has been widely reported that covalently connected 2D materials, including graphene [230], h-BN [231], and MXene [45] are known for their good mechanical properties and electrochemical performance. For instance, robust graphene oxide backbones were introduced into commercial polypropylene (PP) separators, which greatly improved the mechanical properties of the separator and blocked lithium dendrite growth (Fig. 13b) [232]. Meanwhile, polyacrylamide chains with a large number of polar groups were integrated on the graphene oxide surface, which enables homogeneous and fast lithium ionic flux on the surface of Li metal. Although monolayer h-BN possesses exceptional in-plane mechanical strength with a Young’s modulus approaching ~ 1.0 TPa, its practical application is often limited by the presence of intrinsic defects, such as cracks and wrinkles, which can compromise its protective capability. Xie et al. prepared chemically and mechanically stable hybrid LiF/h-BN films, which suppress lithium dendrite formation from chemical and physical properties, thereby enabling more than 300 cycles at high Coulombic efficiency [46]. Considering the high impedance and poor interfacial contact for the graphene, composite, and ceramic-based protective layers, Cha et al. created a protective barrier between Li metal and electrolyte with atomic MoS_2_ layers (Fig. 13c) [233]. The results display that a large amount of Li atoms can intercalate into the MoS_2_ layer structure to reduce the interfacial resistance and facilitate a consistent flow of Li^+^ into and out of bulk Li metal.

Battery thermal stability is another important design consideration that is shown to be improved by the proper choice of 2D materials. Polyolefin-type separators have been successfully used commercially for their high electrochemical stability. However, the low melting temperature of polyolefin, for example, 165 °C for PP, limits their further application in operation situation [44]. Integrating the 2D materials into the polyolefin-type separator can greatly increase the thermal stability of separator [234]. In addition, the high thermal conductivity of 2D materials, such as BN and graphene displays quick responsive capable of inhibiting the growth of lithium dendrite and homogenize thermal propagation [231, 235, 236]. Han et al. conducted graphene-coated separator to eliminate the local hot spots, which come from the accumulated overpotential heat and poor local thermal diffusion [235]. The graphene layer affords timely diffusion of local heat generated by irregular Li growth and incipient dendrite formation, achieving the stable and uniform lithium deposition to deter further degradation (Fig. 13d).

### Electrochemical Stability

Stable reaction interfaces and environments play a pivotal role in the electrochemical reaction for uniform lithium stripping and deposition. For active lithium metal, an artificial solid–electrolyte interface (SEI) can be well designed by 2D materials, e.g., phosphorene [237]. SnS_2_ [238], and fluorinated graphene [74]. The phosphorene-derived Li_3_P layer avoids the decomposition of electrolyte for its high redox potential and realizes stable cycle performance over 500 cycles in Li symmetric cells [237]. SnS_2_ will react spontaneously with Li metal and form a robust multifunctional SEI layer consisting of Li_5_Sn_2_, Li_7_Sn_2_ phases with high Li-ion diffusion ability and superior Li affinity, and the Li_2_S phase can serve as blocking shield of electrons at the SEI/Li interface [238]. Consequently, the Li symmetric cells exhibit a stable cycling behavior of over 2700 h 1 mA cm^−2^ for 1 mAh cm^−2^. Wang et al. designed a composite separator consisting of covalent organic framework (COF) nanosheets anchored onto cellulose nanofibers and integrated on PP separator. The 2D COF with abundant functional groups can simultaneously modulate Li-ion transport with high Li^+^ transference number about 0.65 mS cm^−1^ and robust SEI film components [47]. It is also identified that Li metal can effectively regulate Li⁺ transport, achieving a high Li⁺ transference number (~ 0.65) and promoting the formation of a stable SEI [239]. While soluble redox mediators (RMs) have been proved to be efficient in promoting the formation/decomposition of Li_2_O_2_ products, the shuttle effects of RMs across the separator accelerate the corrosion of Li metal and growth of Li dendrites. 2D materials were also leveraged to block the migration of RM. Shi et al. designed a chemical binding strategy based on Ti_3_C_2_ MXene modified separator to suppress the I_3_^−^ shuttling in LiI-involved LOBs [166]. The results show that –OH terminal groups on MXene functioned as effective binding sites for suppressing the migration of I_3_^−^, which increases the cycle stability by three times in LOBs.

### Expedite and Homogenize the Lithium-Ion Flux

Regulation of lithium-ion flux in transport rate and homogeneity has obvious advantages when compared with other strategies in enhancing the reaction dynamics and preventing the growth of lithium dendrites. MXene which has abundant surface functional groups, especially fluorinated terminals, tends to generate uniform, dense, and durable protection layer on the anode surface, effectively homogenizing the lithium flux and inducing uniform nucleation [43]. Wang et al. verified that fluorinated functional groups on Ti_3_C_2_T_x_ MXene could induce uniform nucleation of lithium. The protected Li anode displayed high stability for over 1300 h at 0.5 mA cm^−2^, which is about 3 times longer than the pristine Li anode (Fig. 13e) [240]. Ti_3_C_2_T_x_ MXene was also introduced into mixing with solid-state electrolyte, in which the O and F groups provide more plating sites and lower nucleation energy [48]. As a result, the arrangement of Li atoms inherits the atomic structure of MXene with sheet-like structure and significantly suppresses the formation of dendritic Li. There are two paths for the penetration of lithium ions through the 2D materials: migration through gaps between the layers or diffusion through the pore structure directly. With atomic thickness, 2D materials can be assembled into ultrathin membranes, thereby minimizing the transport resistance to maximize the lithium-ion flux permeation. Meanwhile, uniform chemistry also reduces the diffusion barrier on the surface. For instance, Li et al. found that lithium-ion diffusion coefficient increased with the reduction of Ti_3_C_2_T_x_ MXene layers [241]. Integrating the CNT into the MXene interface can disrupt the alignment and promote the surface opening and internal expansion, realizing larger and faster ion transport channels [178]. In addition, Xiong et al. reported that the sub-nanometer pores at the Ti vacancies of ultrathin Ti_0.87_O_2_ nanosheets provide fast pathways for the diffusion of lithium ions [242]. In summary, 2D materials play a key role across the anode, separator, and electrolyte components in LOBs. Their excellent mechanical robustness enables effective suppression of lithium dendrite growth, thereby enhancing cycling stability and safety. In addition, transition metal sulfides can exhibit favorable interfacial compatibility with Li metal, which helps reduce interfacial resistance and accelerate charge transfer kinetics. Among various 2D materials, MXenes stand out as one of the most extensively studied systems due to their metallic conductivity and abundant surface functional groups, enabling efficient electron transport, interfacial regulation, and homogenized ion flux. Overall, a fundamental understanding of the physicochemical properties of 2D materials is essential for guiding the rational design and performance optimization of LOBs systems.

## Summary and Perspective

This review systematically summarizes the recent progress of 2D materials applied in LOBs, focusing on engineering strategies, unique structural anisotropy, and performance relationship for accelerating ORR and OER processes. In particular, the catalytic properties of 2D materials arising from surface planes and edge sites are discussed in the context of structural engineering at the point, plane, and bulk levels. These endow 2D materials with large surface areas, abundant active sites, and tunable electronic structures, collectively enhancing catalytic activity, reducing overpotentials, and improving reaction reversibility. In addition, 2D materials also exhibit great potential in other components, such as lithium anode protection, electrolyte stabilization, and fast lithium-ion transport, thereby enabling the construction of more efficient and stable LOBs. Despite these advances, several fundamental challenges remain in the controlled structural design, mechanistic understanding, and integrated optimization of 2D materials, which require further exploration and breakthroughs. Meanwhile, Table 2 systematically summarizes the advantages, challenges, and opportunities of 2D materials.

**Table 2:** Advantages, challenges, and opportunities for 2D materials

2D materials	Advantage	Challenge	Opportunities
Graphene-based	High electronic conductivity; large specific surface area; good modulation substrate; good flexibility	Inert intrinsic catalytic activity; weak adsorption strength toward reaction species; difficult recognition and stabilize of active sites	Pricing heteroatom doping; topological-defect-rich structures; graphene shell encapsulating catalysts;
Transition Metal oxide	High catalytic activity; multiple valence states chemistry; good stability to oxygen intermediate	Low electronic conductivity; catalyst center designment; balanced catalytic activity toward reversible reaction	Ultrathin nanosheets designing; vacancy engineering; heterostructure construction
Transition Metal hydroxides	Large and tunable interlayer spacing; abundant active sites; facile ion transport; structural flexibility	Limited conductivity; low structure stability; prices control of metal cation distribution	Interlayer/intralayer engineering; constructions of ultrathin superlattices; multimetallic composition modulation
MXene-based	High metallic conductivity; uniform morphology; rich species	Surface termination modulation; low structure and chemical stability; high-quality and scalable exfoliation	Surface termination engineering; defect engineering; exploration of theoretical predicted composition
Transition metal dichalcogenides	High catalytic activity; hierarchical morphologies; different coordination patterns	Large-scale synthesis of ultrathin nanosheets; limited active sites exposure; low electronic conductivity	Liquid exfoliation and activation; phase engineering; edge-site engineering; lateral/vertical heterostructure construction

First, exploring the synergistic roles of 2D materials within the whole battery system, e.g., cathode, electrolyte, and anode, is a promising route to achieve enhanced performance. Owing to their mechanical robustness and favorable interfacial chemistry, 2D materials have also shown great potential in lithium anode protection and in facilitating rapid lithium-ion transport. These materials can effectively suppress dendrite growth, stabilize the solid–electrolyte interphase, and ensure uniform ion flux across interfaces. The desirable paths should emphasize coordinated design of cathode, anode, and electrolyte to achieve functional complementarity and interfacial compatibility, thus enabling low overpotentials and high reversibility even under high-rate operating conditions.

Second, controllable structure regulation and precise characterization are essential to build structure–performance relationships. Given that strategies such as defect engineering, heteroatom doping, heterostructure construction, and surface functionalization can effectively tune electron density and orbital occupation, they provide approaches for optimizing the adsorption strength and reaction pathways, reducing overpotentials, and improving the kinetics of product formation and decomposition. It is feasible to identify the intrinsic catalytic performance governed by atomic configuration and electronic structure through advanced experimental techniques. Although some precise methods, such as chemical vapor deposition and plasma-assisted decoration, have prepared well-defined 2D materials on specific substrates [243, 244], especially for semiconductor device applications, such approaches do not retain the advantage of large-scale production inherent to conventional chemical processes and thus remain challenging to meet the catalyst mass loading requirements in LOBs.

Third, catalytic performance is subject to the dynamic behavior of active sites and the evolution of products. It remains an urgent task to conduct in-operando characterizations using cutting-edge experimental techniques (e.g., in situ Raman spectroscopy, electrochemical mass spectrometry, and multiscale simulations), which enable real-time tracking of product formation and migration, providing mechanistic insight into discharge product evolution and energy barrier modulation at the atomic and electronic levels. In particular, clarifying the reaction pathways and deactivation mechanisms of 2D materials during ORR/OER will provide critical insights for connecting their electrochemical performance with atomic structure and catalytic mechanisms.

Finally, the transition from Li–O_2_ battery to Li–air battery presents a core challenge in the complex chemistry of the system under ambient air. Components such as CO_2_ and H_2_O in ambient air can introduce additional reaction pathways alongside the intrinsic Li–O_2_ mechanism, leading to side reactions like Li–CO_2_ and Li–H_2_O pathways. The formation of by-products such as Li_2_CO_3_ and LiOH further passivates electrodes and reduces reversibility and lifespan of batteries. Elucidating the formation/decomposition pathways of Li_2_O_2_, as well as understanding the impact of impurities like Li_2_CO_3_ and LiOH, is crucial for guiding the design of both catalysts and the overall system. Despite these challenges, the performance enhancement of Li–air batteries still primarily relies on system-level design strategies from the aspects of preventing the side reactions to purify the Li–O_2_ pathway. Beyond the design of efficient 2D cathode catalysts, common approaches include Li anode protection, the use of novel electrolytes (e.g., ionic liquids), solid-state electrolytes, gas-selective membranes, and redox mediators.

In summary, 2D materials, with their tunable structures, controllable electronic states, and versatile functionalities, show great potential for advancing LOBs. Future research should take an integrated strategy that combines rational structural design, clear mechanistic understanding, and systems engineering to advance overall performance and fundamental understanding. Such endeavors will pave the way toward efficient, stable, and rechargeable LOBs, accelerating their transition from laboratory research to practical application and contributing to the development of next-generation energy storage technologies.
